# Synergistic Solvation Strategy for Low-Temperature Alkaline Zinc−Ferricyanide Flow Battery

**DOI:** 10.34133/research.1118

**Published:** 2026-02-02

**Authors:** Yalu Xin, Chen Li, Wei Gao, Yongping Chen

**Affiliations:** ^1^ Key Laboratory of Energy Thermal Conversion and Control of Ministry of Education, School of Energy and Environment, Southeast University, Nanjing, Jiangsu 210096, P. R. China.; ^2^ Jiangsu Key Laboratory of Micro and Nano Heat Fluid Flow Technology and Energy Application, School of Environmental Science and Engineering, Suzhou University of Science and Technology, Suzhou, Jiangsu 215009, P. R. China.

## Abstract

Alkaline zinc–ferricyanide flow batteries (AZFFBs) emerge as promising candidates for long-duration energy storage. However, at cryogenic temperatures, these systems suffer from electrolyte solidification, anodic zinc dendrite formation, zinc-related side reactions, and cathodic Fe(CN)_6_^4−^ precipitation-induced capacity decay. Herein, we propose a synergistic solvation strategy in which Li^+^ and Cl^−^ jointly inhibit the formation of tetrahedral hydrogen bond networks, thereby lowering the liquid–solid transition peak temperature of both the anolyte and catholyte. Meanwhile, Cl^−^ is utilized to construct a water-poor solvation structure around Zn(OH)_4_^2−^ to optimize zinc deposition and inhibit the side reactions, while Li^+^ enhances the solubility of Fe(CN)_6_^4−^ by incorporating additional water molecules into its solvation structure through strong ion–dipole interactions. The optimized AZFFB exhibits outstanding low-temperature performance, achieving stable cycling at −20 °C with an average coulombic efficiency of 99.54%. It also demonstrates excellent stability at room temperature, sustaining over 500 cycles at 28 °C with an average coulombic efficiency of 99.79%, representing more than a 22-fold extension in cycle life. Additionally, the AZFFB exhibits robust stability under fluctuating temperature conditions. These breakthroughs markedly enhance the potential of AZFFBs as viable solutions for extreme-environment energy storage, particularly in polar region microgrids, cold-climate off-grid power systems, and subsea power applications.

## Introduction

The growing need for long-duration energy storage has become increasingly apparent due to the intermittency of renewable energy sources, such as solar and wind power, and the resulting demand for enhanced power system stability and reliability [1–3]. Aqueous redox flow batteries offer a promising solution for long-duration energy storage by providing high efficiency, extended lifespan, exceptional safety, and the advantage of power/capacity decoupling [4–14]. Among these, alkaline zinc–ferricyanide flow batteries (AZFFBs) stand out as optimal candidates for large-scale applications owing to their high electrochemical activity, favorable kinetics, and low cost [15–20]. To enhance the cycling stability of the system, substantial efforts have focused on mitigating zinc dendrite growth and suppressing active-species crossover [21–23]. Strategies to increase ferricyanide solubility have also been widely explored to expand the practical capacity [24–26]. However, these strategies alone are insufficient to ensure robust operation across a broad range of operating conditions. Among them, electrolyte freezing and ferricyanide precipitation at low temperatures markedly constrain the operational range and cycling stability of the system [27–29]. These challenges hinder their deployment in extreme environments such as polar regions, off-grid cold-climate systems, and deep-sea applications, highlighting the urgent need for innovative strategies to develop AZFFBs capable of functioning at low temperatures.

At low temperatures, AZFFBs experience increased hydrogen bonding (HB) among water molecules in the electrolyte, transitioning from initially disordered small water clusters to long-range-ordered HB networks, which ultimately leads to electrolyte freezing [30–32]. To address this, researchers have explored various strategies, including incorporating organic cosolvents, adding chemical additives, and leveraging ion effects, to reconstruct the large HB network, inhibit the formation of ordered tetrahedral structures, and markedly lower the liquid–solid transition peak temperature (*T*_t_) of the electrolyte [31,33–36]. Among these approaches, organic antifreeze additives can effectively lower the freezing point, but their high cost, poor chemical stability, and strong interfacial adsorption that occupies electroactive sites and hinders Zn^2+^ transport often limit their practical application [37–39]. Despite these efforts, achieving a low *T*_t_ alone is insufficient to ensure stable and efficient AZFFB operation at low temperatures. On the one hand, low temperatures exacerbate the nonuniform diffusion of ions in the electrolyte, resulting in uneven zinc deposition on the anode [34,40–42]. On the other hand, weakened ion–dipole interactions between Fe(CN)_6_^4−^ and water molecules, coupled with enhanced HB among water molecules, reduce the solubility of Fe(CN)_6_^4−^, leading to its precipitation and potential channel blockage [43,44]. Unfortunately, few studies have successfully achieved simultaneous reduction of *T*_t_, mitigation of zinc-related side reactions, and suppression of cathodic Fe(CN)_6_^4−^ precipitation. Reconciling these factors to enable high-performance AZFFBs to operate stably under low-temperature conditions remains an important challenge.

**Fig. 1.**
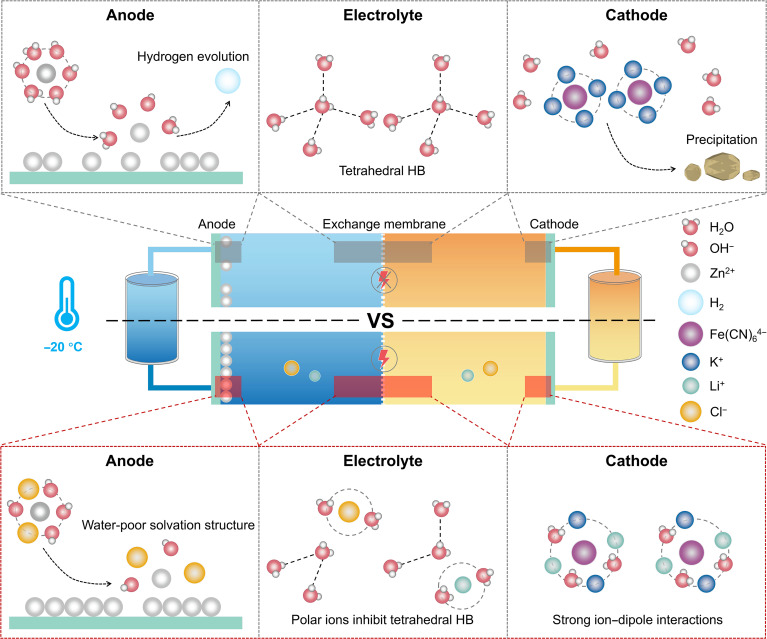
Schematic illustration of the synergistic solvation strategy for alkaline zinc–ferricyanide flow batteries (AZFFBs). The strategy aims to simultaneously reduce *T*_t_, optimize zinc deposition, inhibit zinc-related side reactions, and suppress cathodic Fe(CN)_6_^4−^ precipitation. HB, hydrogen bonding.

To tackle this issue, we present a synergistic solvation strategy that simultaneously enhances the antifreezing performance of both the anolyte and catholyte, enabling stable operation at temperatures as low as −20 °C, the lowest temperature reported for AZFFBs to date. This strategy applies the polar ions Li^+^ and Cl^−^ to effectively disrupt tetrahedral HB networks among water molecules, thereby lowering *T*_t_ while simultaneously modifying the solvation structures of Zn(OH)_4_^2−^ and Fe(CN)_6_^4−^ (Fig. 1). On the anode side, Cl^−^ strongly interacts with Zn(OH)_4_^2−^, partially replacing water molecules in its solvation shell and constructing a water-poor solvation structure to optimize zinc deposition and inhibit side reactions. On the cathode side, the small ionic radius of Li^+^ allows it to partially replace K^+^ around Fe(CN)_6_^4−^, strengthening ion–dipole interactions and incorporating additional water molecules into its solvation structure, thereby markedly enhancing solubility at low temperatures. As a result, the LiCl-containing AZFFB exhibits remarkably low-temperature performance, maintaining stable operation at −20 °C for 150 cycles at a current density of 20 mA cm^−2^ with average coulombic efficiency (CE) and energy efficiency (EE) values of 99.54% and 74.87%, respectively. Notably, it also demonstrates excellent stability at 28 °C, achieving 500 cycles at a current density of 40 mA cm^−2^ with average CE of 99.79% and EE of 86.84%.

## Results and Discussion

### Design of the synergistic solvation strategy

To overcome the persistent challenges of electrolyte freezing, anode side reactions, and cathode active material precipitation in AZFFBs under low temperatures, this study developed a synergistic solvation strategy that addresses key processes, including HB network reconstruction, Zn(OH)_4_^2−^ solvation structure modulation, and enhanced dissolution of Fe(CN)_6_^4−^. This strategy employs a Li^+^/Cl^−^ dual-ion synergistic design to engineer polar-ion-mediated HB reconstruction, thereby achieving concurrent electrolyte stabilization and interfacial side reaction inhibition while demonstrating unprecedented cyclability at −20 °C (Fig. 1).

**Fig. 2.**
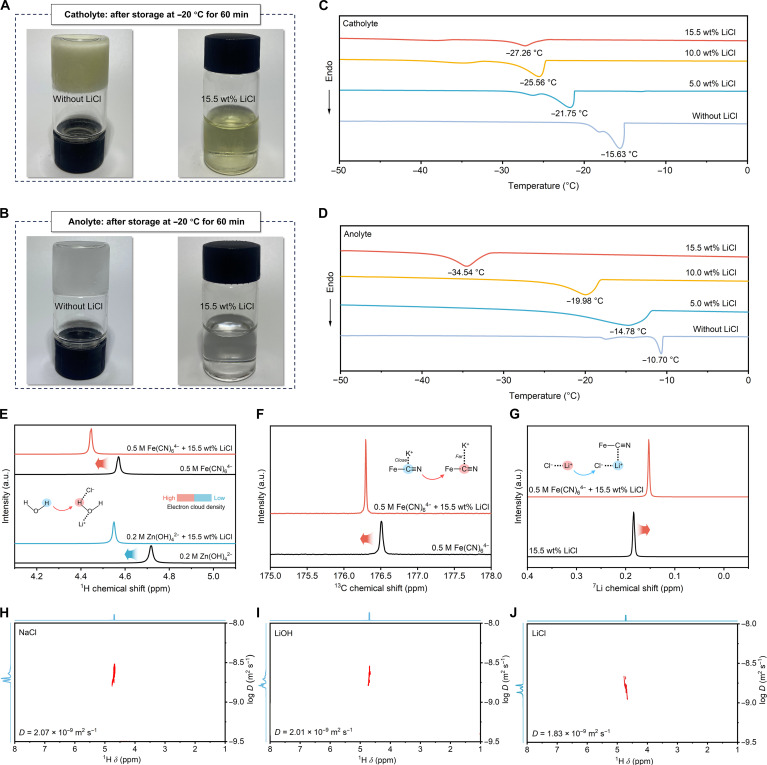
Mechanistic investigation of Li^+^ and Cl^−^ enhanced the antifreezing characteristics of AZFFB electrolytes. (A) Optical images of catholytes with and without LiCl at −20 °C. (B) Optical images of anolytes with and without LiCl at −20 °C. (C) The *T*_t_ evolution of the catholyte with increasing LiCl concentration. (D) The *T*_t_ evolution of the anolyte with increasing LiCl concentration. (E) ^1^H nuclear magnetic resonance (NMR) spectra illustrating the influence of Li^+^ and Cl^−^ on the electron cloud density around the H atoms of water molecules. (F) ^13^C NMR spectra demonstrating the effect of Li^+^ on the C≡N chemical shift of Fe(CN)_6_^4−^ in the catholyte. (G) ^7^Li NMR spectra evidence for electron cloud density redistribution around Li^+^ in the catholyte. (H) ^1^H DOSY spectra of water molecules in 1 M NaCl. (I) ^1^H DOSY spectra of water molecules in 1 M LiOH. (J) ^1^H diffusion-ordered spectroscopy (DOSY) spectra of water molecules in 1 M LiCl.

Visualization and differential scanning calorimetry (DSC) were employed to elucidate the effects of Li^+^ and Cl^−^ concentrations on the low-temperature stability of alkaline zinc–ferricyanide electrolytes. At −20 °C, optical images clearly reveal that LiCl-containing electrolytes remain unfrozen, whereas the pristine catholyte and anolyte without LiCl undergo visible freezing (Fig. 2A and B). This macroscopic observation indicates that the addition of LiCl markedly improves the low-temperature stability of the electrolytes. DSC measurements provide further insight: The incorporation of Li^+^ and Cl^−^ ions markedly reduces *T*_t_. When the LiCl concentration reaches 15.5 wt%, the *T*_t_ values during freezing decrease to −27.26 and −34.54 °C for the catholyte and anolyte, respectively (Fig. 2C and D and Figs. S1 and S2). Extended measurements confirm that, if necessary, subzero stability can be achieved by increasing the LiCl concentration to achieve even lower *T*_t_ values, particularly for the anolyte (Fig. S3). Moreover, optical images demonstrate that higher LiCl concentrations further reduce the *T*_t_ of both the catholyte and anolyte, consistent with DSC measurements, while temperature reduction induces Fe(CN)_6_^4−^ to precipitate in a LiCl-free catholyte, whereas LiCl-containing electrolytes effectively suppress such precipitation even at low temperatures (Figs. S4 and S5). It is worth noting that although the DSC results show only a small difference in catholyte *T*_t_ between 10 and 15.5 wt% LiCl, the freezing behaviors differ markedly under practical operating conditions. In a real flow battery, the large volumes of flowing electrolytes and abundant heterogeneous interfaces promote ice nucleation [45,46]. Consequently, the 10 wt% catholyte freezes at −20 °C, whereas the 15.5 wt% catholyte remains fully liquid and supports stable cycling. In addition, DSC measurements indicate that while the anolyte achieves a *T*_t_ comparable to that of the 15.5 wt% LiCl catholyte at a lower concentration of around 12 wt%, the catholyte requires 15.5 wt% to remain stable (Fig. S6). To avoid persistent concentration gradients and disrupt osmotic and compositional balance, 15.5 wt% was adopted for both electrolytes.

The Li^+^ and Cl^−^ effectively decrease *T*_t_, suppress crystallization, and stabilize Fe(CN)_6_^4−^ dissolution in a cryogenic environment, which is attributed to polar-ion-mediated HB reconstruction. To probe the roles of Li^+^ and Cl^−^ in modifying solvation structures, ^1^H nuclear magnetic resonance (NMR) spectra were analyzed. The ^1^H NMR spectra demonstrate that adding Li^+^ and Cl^−^ causes the chemical shifts of both catholyte and anolyte toward higher fields, indicating an increased electron cloud density around H atoms in water molecules (Fig. 2E). Mechanistically, Li^+^ interacts with the negatively charged O atoms of water molecules, reducing the electron cloud density around adjacent H atoms, thereby shifting their chemical shifts to a lower field. Conversely, Cl^−^ binds to the positively charged H atoms, enhancing the electron cloud density around H atoms, which causes the chemical shift to move to higher fields. The stronger ion–dipole interaction between Cl^−^ and water molecules compared to that of Li^+^ ultimately dominates the overall upfield shift, resulting in a net increase in hydrogen electron density [47,48]. The ion–dipole interactions between polar ions (Li^+^ and Cl^−^) and water molecules disrupt the tetrahedral HB arrangement, thereby elevating configurational entropy. This entropy-driven disorder suppresses the formation of ordered icelike structures, which underlie the cryoprotective functionality of the electrolyte.

The ^13^C NMR spectra demonstrate that the chemical shift moves toward a higher field after adding LiCl into the catholyte, reflecting an increased electron cloud density around the C atom (Fig. 2F). This finding suggests that the presence of Li^+^ inhibits the ionic interaction between K^+^ and Fe(CN)_6_^4−^. The ^7^Li NMR spectra demonstrate that the chemical shift moves toward a higher field after adding the catholyte to the LiCl solution, indicating an increased electron cloud density around Li^+^ (Fig. 2G). This result implies that Li^+^ integrates into the solvation structure of Fe(CN)_6_^4−^ and partially replaces K^+^, thereby strengthening the ion–dipole interactions between Fe(CN)_6_^4−^ and water molecules. These enhanced interactions facilitate the incorporation of additional water molecules into the solvation structure of Fe(CN)_6_^4−^, effectively preventing its aggregation and markedly improving its low-temperature solubility.

To elucidate the synergistic effect of Li^+^ and Cl^−^ on the HB configuration of water molecules, ^1^H diffusion-ordered spectroscopy (DOSY) measurements were performed on LiOH and NaCl aqueous solutions, which contain different anions and cations, respectively, in comparison with LiCl. At identical ion concentrations (1 M), the LiCl solution exhibited the lowest diffusion coefficient (1.83 × 10^−9^ m^2^ s^−1^) of water molecules, in contrast to the higher values observed for NaCl and LiOH solutions (Fig. 2H to J). The DSC measurements of the 3 aqueous solutions further corroborate that the synergistic interaction between Li^+^ and Cl^−^ enables a substantially lower *T*_t_ compared with those of LiOH and NaCl aqueous solutions (Fig. S7). This highlights the cooperative effect of Li^+^ and Cl^−^ in restructuring the HB network, enabling more effective suppression of tetrahedral configurations under low-temperature conditions.

### Effective reconstruction of the solvation structure

To gain deeper insight into the mechanism by which LiCl enhances the low-temperature stability of alkaline zinc–ferricyanide electrolytes, the solvation structures of the catholyte and anolyte were systematically investigated.

**Fig. 3.**
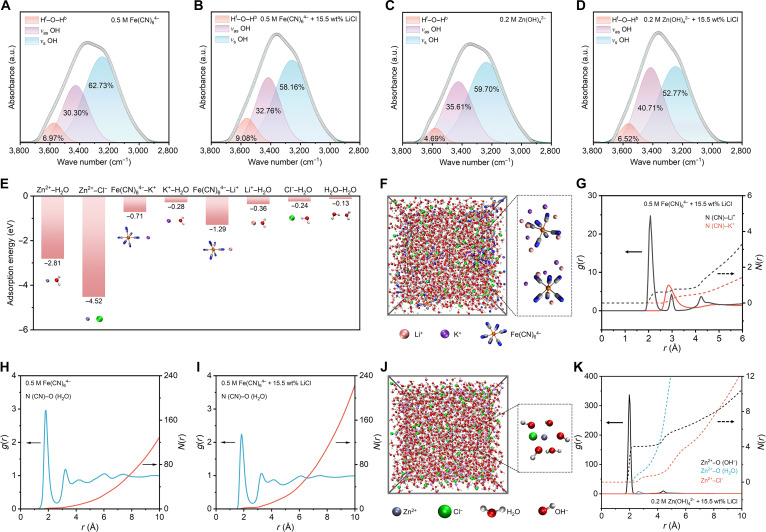
Verification of the effective reconstruction of the solvation structure in the electrolyte of AZFFBs. (A) Proportions of 3 subpeaks in the Fourier transform infrared spectroscopy (FTIR) water spectrum of the catholyte without Li^+^ and Cl^−^. (B) Proportions of 3 subpeaks in the FTIR water spectrum of the catholyte with Li^+^ and Cl^−^. (C) Proportions of 3 subpeaks in the FTIR water spectrum of the anolyte without Li^+^ and Cl^−^. (D) Proportions of 3 subpeaks in the FTIR water spectrum of the anolyte with Li^+^ and Cl^−^. (E) Adsorption energies of different substances in the anolyte and catholyte. (F) Molecular dynamics (MD) simulation of the catholyte containing Li^+^. (G) Radial distribution functions of N (CN)–Li^+^ and N (CN)–K^+^ in the catholyte containing Li^+^. (H) Radial distribution function of N (CN)–O (H_2_O) in the catholyte without Li^+^. (I) Radial distribution function of N (CN)–O (H_2_O) in the catholyte containing Li^+^. (J) MD simulation of the anolyte containing Cl^−^. (K) Radial distribution functions of Zn^2+^–O (OH^−^), Zn^2+^–O (H_2_O), and Zn^2+^–Cl^−^ in the anolyte containing Cl^−^.

Fourier transform infrared spectroscopy (FTIR) and Raman spectra were performed on the catholyte and anolyte to verify the influence of Li^+^ and Cl^−^ on the HB configuration of the water molecule. The results show that Li^+^ and Cl^−^ cause a shift in water peaks in both FTIR and Raman spectra (Figs. S8 to S11). To better analyze the impact of water peak shifts on antifreeze performance, the water peaks obtained from FTIR were categorized into 3 subpeaks based on different HB configurations: *v*_s_ OH, *v*_as_ OH, and H^f^–O–H^b^ stretching peaks (Fig. 3A to D). The *v*_s_ OH stretching peak appears around 3,250 cm^−1^, representing the HB network of water molecules in a regular tetrahedral configuration. The *v*_as_ OH and H^f^–O–H^b^ stretching peaks, centered around 3,400 and 3,600 cm^−1^, respectively, represent the HB network of water molecules with incomplete coordination [31]. A higher proportion of *v*_as_ OH and H^f^–O–H^b^ stretching peaks correlates with a lower likelihood that the electrolyte will freeze. The FTIR spectra indicate that the addition of 15.5 wt% LiCl increases the proportion of incompletely coordinated HB in water molecules of the catholyte by 4.57% (Fig. 3A and B) and in the anolyte by 6.93% (Fig. 3C and D).

Adsorption energy analysis reveals that the interaction between Zn^2+^ and Cl^−^ is markedly stronger than that between Zn^2+^ and H_2_O, indicating that Cl^−^ can displace water molecules in the solvation shell of Zn(OH)_4_^2−^ (Fig. 3E and Note S1). The adsorption energy of Fe(CN)_6_^4−^ with Li^+^ is much higher than that with K^+^, while the Li^+^–H_2_O interaction exceeds K^+^–H_2_O. This suggests that Li^+^ not only replaces partial K^+^ around Fe(CN)_6_^4−^ but also enhances the water molecule content in its solvation shell. Additionally, comparative adsorption energy data confirm that both Li^+^ and Cl^−^ inhibit the formation of HB among water molecules. Molecular dynamics simulations elucidate the reconstruction of the solvation structure of Fe(CN)_6_^4−^ and Zn(OH)_4_^2−^ induced by Li^+^ and Cl^−^ (Fig. 3F to K, Table S1, and Note S2). Radial distribution function analysis shows that in the catholyte containing Li^+^ and Cl^−^, 3 characteristic Li^+^ peaks appear at 2.1, 3.0, and 4.2 Å from the N atom (Fig. 3F and G). Within 6 Å of the N atom in Fe(CN)_6_^4−^, the average coordination numbers of K^+^ and Li^+^ are 1.45 and 3.33, respectively. This suggests that Li^+^ can be integrated into the solvation structure of Fe(CN)_6_^4−^. By comparing the intensities of the N (CN)–K^+^ characteristic peaks in the presence and absence of LiCl, it is confirmed that Li^+^ markedly weakens the adsorption strength between K^+^ and Fe(CN)_6_^4−^ (Figs. S12 and S13). Furthermore, radial distribution function analysis of water molecules around the N atom confirms that adding LiCl markedly increases the number of water molecules surrounding Fe(CN)_6_^4−^ (Fig. 3H and I and Table S2). Molecular dynamics simulations of the anolyte indicate that Cl^−^ can replace some water molecules in the solvation structure of Zn(OH)_4_^2−^, which helps reduce the number of active water molecules during desolvation, thereby inhibiting the hydrogen evolution reaction (Fig. 3J) [49–51]. Based on radial distribution functions, within 3 Å of Zn^2+^, the average coordination numbers of OH^−^, H_2_O, and Cl^−^ are 4.05, 1.31, and 0.70, respectively (Fig. 3K). These simulation results can strongly support the experimental data.

### Electrochemical stability of electrodes

**Fig. 4.**
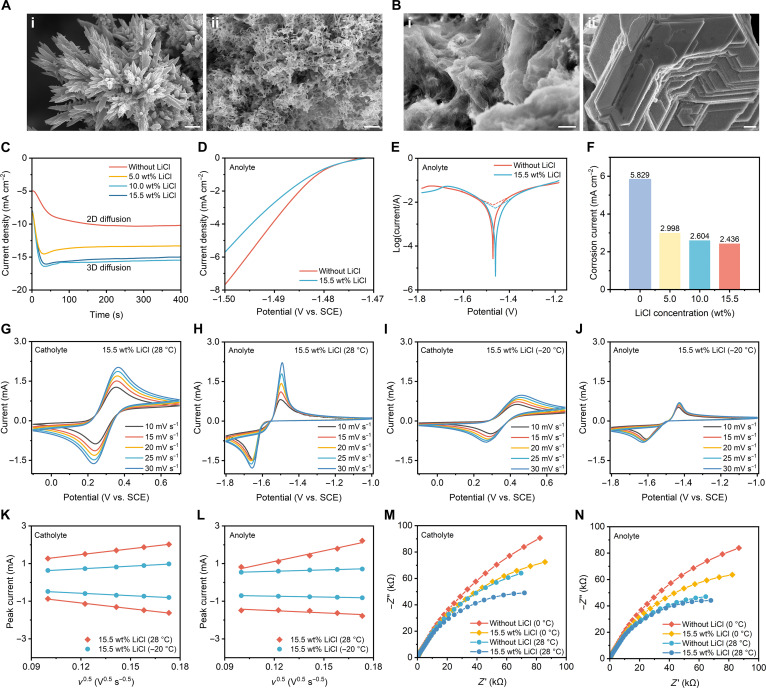
The influence of LiCl on cathode and anode stability. (A) Scanning electron microscopy (SEM) images of zinc deposited on the surface of the graphite felt electrode at 10 mAh cm^−2^ areal capacity and 10 mA cm^−2^ current density in the anolyte without LiCl at 28 and 0 °C. Scale bar: 2 μm. (B) SEM images of zinc deposited on the surface of the graphite felt electrode at 10 mAh cm^−2^ areal capacity and 10 mA cm^−2^ current density in the anolyte with 15.5 wt% LiCl at 28 and 0 °C. Scale bar: 2 μm. (C) Chronoamperometric (CA) curves of the anolyte with increasing LiCl concentrations at a constant overpotential of −150 mV. (D) Linear sweep voltammetry (LSV) curves of the anolyte with and without LiCl. (E) Tafel curves of the anolyte with and without LiCl. (F) The corrosion current evolution of anolyte with increasing LiCl concentration. (G) Cyclic voltammetry (CV) curves of the catholyte at varying scan rates at 28 °C. (H) CV curves of the anolyte at varying scan rates at 28 °C. (I) CV curves of the catholyte at varying scan rates at −20 °C. (J) CV curves of the anolyte at varying scan rates at −20 °C. (K) Linear corrections of the LiCl-containing catholyte at 28 and −20 °C between the peak currents from oxidation and reduction processes and the square root of the scan rate. (L) Linear corrections of the LiCl-containing anolyte at 28 and −20 °C between the peak currents from oxidation and reduction processes and the square root of the scan rate. (M) Electrochemical impedance spectroscopy (EIS) of the catholyte with different LiCl concentrations. (N) EIS of the anolyte with different LiCl concentrations. 2D, 2-dimensional; 3D, 3-dimensional; SCE, saturated calomel electrode.

The synergistic solvation strategy effectively addresses key challenges in the AZFFB by simultaneously reducing *T*_t_, improving zinc deposition, mitigating zinc-related side reactions, and suppressing cathodic Fe(CN)_6_^4−^ precipitation. This coordinated approach ensures excellent electrochemical stability in both the cathode and anode. The addition of LiCl can markedly optimize zinc deposition, and this effect becomes more pronounced as the temperature decreases (Fig. 4A and B and Figs. S14 to S17). Chronoamperometric (CA) analysis reveals that the zinc deposition behavior varies markedly with LiCl concentration. The electrolyte without LiCl exhibits a low and stable current density corresponding to sluggish 2-dimensional surface-limited nucleation (Fig. 4C). With 5.0 wt% LiCl, the nucleation rate increases moderately, while further increasing the LiCl concentration to 10.0 and 15.5 wt% results in a higher steady-state current density, indicating a transition to diffusion-controlled 3-dimensional zinc growth. These findings demonstrate that LiCl addition effectively promotes 3-dimensional nucleation and enables more uniform zinc deposition [52]. Even at −20 °C, scanning electron microscopy observations confirm that the presence of LiCl enables uniform and compact zinc deposition, demonstrating its robust interfacial stabilization capability under low-temperature conditions (Fig. S18). Linear sweep voltammetry (LSV) measurements demonstrate that the addition of LiCl suppresses the hydrogen evolution reaction on the anode, with the inhibitory effect increasing linearly with LiCl concentration (Fig. 4D and Fig. S19). Tafel curves further confirm that LiCl addition enhances anodic corrosion resistance, indicating suppressed hydrogen evolution and a more favorable competition toward zinc deposition (Fig. 4E and F and Fig. S20).

The cyclic voltammetry (CV) analysis demonstrates that the introduction of Li^+^ and Cl^−^ enables both electrolytes to maintain highly symmetric anodic and cathodic peaks across scan rates of 10 to 30 mV s^−1^ and a wide temperature range from −20 to 28 °C, indicating preserved redox reversibility (Fig. 4G to J). With decreasing temperature, the ion-diffusion rate slows down, causing the peak currents at −20 °C to be markedly reduced, while those at 0 °C fall between the values observed at 28 and −20 °C (Figs. S21 and S22). Meanwhile, quantitative evaluation of the ion-diffusion coefficients reveals the ion transport characteristics under different working conditions (Note S3 and Table S3). At 28 °C, the diffusion coefficients of the catholyte redox couple with LiCl are 9.39 × 10^−7^ and 9.17 × 10^−7^ cm^2^ s^−1^. Under the same ambient temperature, the diffusion coefficients of the anolyte redox couple with LiCl are 2.50 × 10^−6^ and 1.07 × 10^−7^ cm^2^ s^−1^. When the ambient temperature decreases to −20 °C, the diffusion coefficients of the catholyte redox couple with the LiCl are 2.44 × 10^−7^ and 2.03 × 10^−7^ cm^2^ s^−1^, and those of the anolyte redox pairs with the LiCl are 4.96 × 10^−8^ and 2.03 × 10^−8^ cm^2^ s^−1^ (Fig. 4K and L). The decrease in temperature did not cause a sharp drop in the diffusion coefficients of the electrolytes, suggesting that adding Li^+^ and Cl^−^ lowers the electrolyte’s *T*_t_ and enables it to maintain good electrochemical performance under low-temperature conditions. In addition, the incorporation of Li^+^ and Cl^−^ slightly decreases the diffusion coefficients of both the catholyte and anolyte, which can be attributed to the increased electrolyte viscosity caused by the higher salt concentration, thereby enhancing internal friction and hindering ion diffusion (Figs. S23 to S25). Electrochemical impedance spectroscopy (EIS) results show that at 28 °C, adding 15.5 wt% LiCl markedly reduces catholyte impedance, reflecting enhanced ionic conductivity and mitigating interfacial polarization (Fig. 4M and N). At 0 °C, the overall impedance increases markedly due to hindered ion transport. However, LiCl incorporation still alleviates interfacial resistance, underscoring its beneficial effect even under low-temperature conditions.

FTIR analysis was performed on the electrolytes before and after battery cycling and static storage to evaluate the impact of Li^+^ and Cl^−^ on the chemical stability of the catholyte and anolyte. The results demonstrate that no structural degradation occurs in either the reduced-state catholyte or the oxidized-state anolyte after 500 charge–discharge cycles (Fig. S26). The sole detectable change is a weak stretching vibration at ~2,040 cm^−1^ in the cycled anolyte, assigned to the C≡N bond vibration of Fe(CN)_6_^4−^. This phenomenon originates from the intrinsic ion crossover effect of the Nafion proton-exchange membrane, which allows trace Fe(CN)_6_^4−^ migration into the anolyte compartment during operation. In addition, long-term stability tests further confirm the robustness of the electrolyte. When the reduced-state catholyte and oxidized-state anolyte are stored separately for 20 d, FTIR spectra show no compositional changes (Fig. S27). Collectively, these findings validate that LiCl incorporation preserves the chemical stability of both the catholyte and anolyte under operational and storage conditions.

### Long-term cycling performance of AZFFBs at low temperatures

**Fig. 5.**
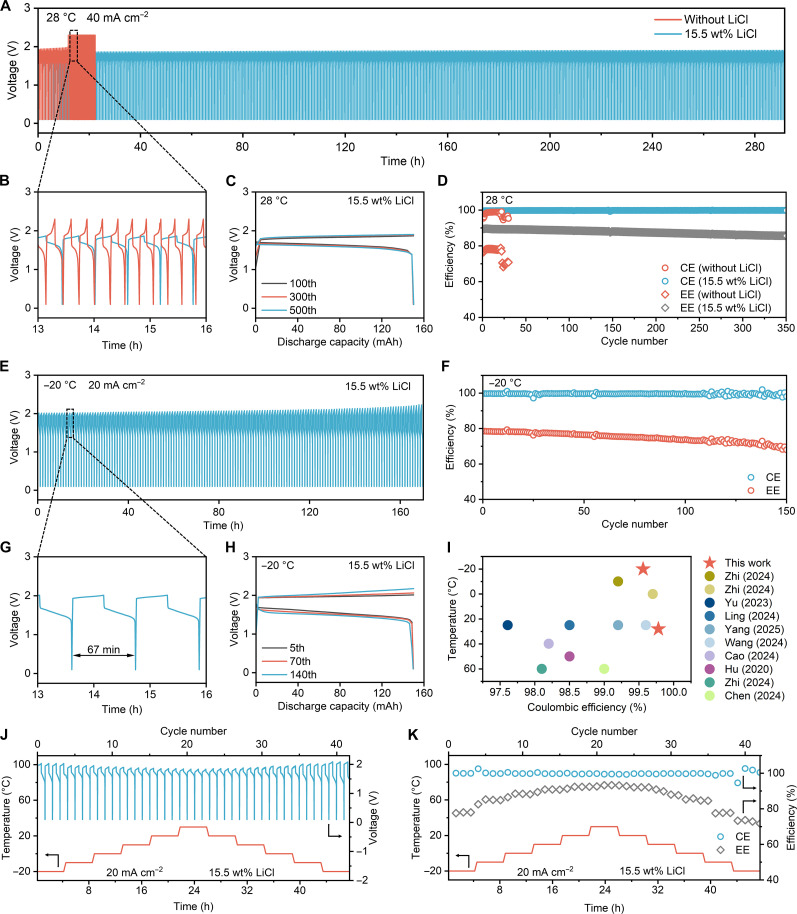
The electrochemical cycling performance of AZFFBs. (A) Voltage curves of the AZFFB during charge–discharge cycling with and without LiCl at 28 °C under a 40 mA cm^−2^ current density. (B) Enlarged view of the voltage curves between 13 and 16 h. (C) Representative voltage curves of the LiCl-containing AZFFB at the 100th, 300th, and 500th cycles. (D) Coulombic efficiency (CE) and energy efficiency (EE) of the AZFFB with and without LiCl at 28 °C under 40 mA cm^−2^ current density. (E) Voltage curves of the LiCl-containing AZFFB during charge–discharge cycling at −20 °C under a 20 mA cm^−2^ current density. (F) CE and EE of the LiCl-containing AZFFB at −20 °C under a 20 mA cm^−2^ current density. (G) Enlarged view of the voltage curves between 13 and 16 h. (H) Representative voltage curves of the LiCl-containing AZFFB at the 5th, 70th, and 140th cycles. (I) Performance comparison of this work with existing research on zinc–ferricyanide flow batteries. (J) Voltage curves of the LiCl-containing AZFFB during charge–discharge cycling under a 20 mA cm^−2^ current density and over a thermal cycling range of −20 to 30 °C. (K) CE and EE evolution of the LiCl-containing AZFFB during −20 to 30 °C thermal cycling under a 20 mA cm^−2^ current density.

To evaluate the temperature adaptability of the synergistic solvation strategy in AZFFBs, cycling performance tests were conducted at both room temperature (28 °C) and low temperature (−20 °C) using battery configurations with an active area of 13.5 cm^2^ (Fig. S28 and Note S3). Precise temperature regulation is achieved through an environmental chamber equipped with proportional–integral–derivative control (Fig. S29). At 28 °C and a current density of 40 mA cm^−2^, the LiCl-containing AZFFB exhibits markedly extended cycling stability compared with the rapid failure of the LiCl-free AZFFB (Fig. 5A and Fig. S30). The enlarged voltage profiles in Fig. 5B highlight the severe polarization in the LiCl-free cell, whereas the LiCl-containing cell maintains stable charge–discharge curves. Moreover, as illustrated in Fig. 5C, the voltage profiles of the LiCl-containing cell at the 100th, 300th, and 500th cycles nearly overlap, confirming its excellent long-term stability. Meanwhile, the average CE and EE of the LiCl-containing AZFFB reach 99.79% and 86.84%, respectively, over the first 500 cycles (Fig. 5D). By contrast, the LiCl-free AZFFB undergoes rapid performance decay under 70% state of charge (SOC) conditions, with voltage polarization emerging from the 22nd cycle and average CE and EE values of 97.66% and 75.82% over the first 30 cycles. The incorporation of LiCl remarkably improves the cycling stability of the AZFFB at room temperature, achieving more than a 22-fold enhancement and substantially increasing CE and EE by 2.13% and 11.02%, respectively. These collective results demonstrate that the synergistic solvation strategy enables room-temperature stability.

For low-temperature validation with a current density of 20 mA cm^−2^ and a charging cutoff condition of 70% SOC, the AZFFB completes 150 cycles while maintaining stable charge–discharge voltages, with average CE and EE values of 99.54% and 74.87%, respectively (Fig. 5E and F and Fig. S31). As shown in Fig. 5G, the duration of a single cycle exceeds 1 h. Moreover, the discharge profiles at the 5th, 70th, and 140th cycles nearly overlap, further demonstrating the excellent reversibility and low polarization of the battery even under low-temperature operation (Fig. 5H). These findings indicate that AZFFB exhibits outstanding electrochemical reversibility and stable energy output with negligible capacity fading, confirming its strong potential for reliable long-term operation even under subzero conditions. In conclusion, these results set new performance benchmarks for AZFFBs in both cycling stability and operational temperature range, markedly outperforming existing studies (Fig. 5I and Table S4) [43,44,53–59].

To investigate the impact of thermal stress in practical AZFFB operation, such as extreme weather, diurnal variations, and load switching, specifically programmed thermal cycling tests were conducted on AZFFBs, simulating temperature transitions from −20 to 30 °C (Note S3). The thermal protocol utilizes a 0.5 °C min^−1^ ramp rate between 10 °C intervals with 4-h stabilization at each temperature node. Progressive temperature increases reduce charge/discharge voltages through accelerated redox reaction kinetics, while subsequent cooling phases gradually restore polarization characteristics (Fig. 5J and Figs. S32 and S33). Throughout thermal cycling, the battery maintains a CE of 99.86% and an EE of 86.01% (Fig. 5K), demonstrating complete electrochemical reversibility under thermal stress. This temperature-independent performance confirms the synergetic solvation strategy’s viability for field deployment in regions with daily temperature fluctuations exceeding 25 °C, without requiring auxiliary thermal regulation systems.

## Conclusion

In this work, we report a synergistic solvation strategy that enables the stable operation of AZFFBs at a low temperature of −20 °C by introducing ions with strong dipole interactions. We employ the synergistic effect of Li^+^ and Cl^−^ to effectively inhibit the formation of tetrahedral hydrogen bond networks while modulating the solvation structures of Fe(CN)_6_^4−^ and Zn(OH)_4_^2−^. Specifically, on the cathode side, Li^+^ partially replaces K^+^ in the solvation structure of Fe(CN)_6_^4−^ and incorporates additional water molecules, markedly suppressing Fe(CN)_6_^4−^ precipitation at low temperatures. On the anode side, Li^+^ disrupts the solvation structure of Zn(OH)_4_^2−^, while Cl^−^ replaces some water molecules within the solvation shell, thereby improving zinc deposition and reducing side reactions. As a result, the optimized AZFFB achieves stable operation at −20 °C with average CE and EE values of 99.54% and 74.87%, respectively. It also demonstrates enhanced cycling stability at room temperature and robust stability under temperature fluctuations. This work provides an effective design strategy for achieving stable performance of redox flow batteries under extreme cold conditions.

## Materials and Methods

### Chemicals and materials

Lithium hydroxide, sodium chloride, sodium hydroxide, and lithium chloride were purchased from Aladdin. Potassium ferrocyanide, potassium ferricyanide, and zinc oxide were purchased from Sigma-Aldrich. The purity of all chemicals and materials reached analytical grade, and they could be directly used in experiments without further purification. The Nafion 115 (Dupont) membrane was purchased from Chemours. All experiments in this work were conducted using deionized water.

### DSC measurements

The phase transition temperature of the electrolyte was measured using a differential scanning calorimeter (DSC8000, Waltham, USA). Firstly, the sample was maintained at 25 °C for 5 min, and then the temperature of the sample was decreased from 25 to −65 °C at a cooling rate of 5 °C min^−1^.

### NMR measurements

NMR analysis of the anolyte and catholyte was conducted using the Bruker Avance III HD 600-MHz spectrometer (Bruker, Germany) to demonstrate the variations of ^1^H, ^7^Li, and ^13^C chemical shifts in various electrolytes at room temperature. A liquid sample was prepared using a coaxial double-tube setup, where the inner tube was loaded with deuterated reagent (D_2_O) and the outer tube contained the target sample.

### ^1^H DOSY measurements

^1^H DOSY spectra were acquired on a Bruker Avance III HD 600-MHz spectrometer (Bruker, Germany). Electrolyte samples were prepared in deuterated reagent (D_2_O) and measured at 28 °C.

### FTIR and Raman spectroscopy measurements

At room temperature, the FTIR spectra of the electrolytes were recorded using a Fourier transform infrared spectrometer (Thermo Nicolet iS50, Waltham, USA), and the Raman spectra were obtained with a micro-Raman spectrometer (HORIBA France SAS XploRA, Palaiseau, France). For the FTIR comparison before and after cycling, the post-cycled electrolyte was collected from the AZFFB experiment carried out at 28 °C.

### CA, LSV, Tafel, CV, and EIS measurements

All electrochemical measurements were conducted on an electrochemical workstation (CHI700E, Shanghai, China). CA was performed using a 2-electrode configuration, whereas LSV, Tafel, CV, and EIS measurements were carried out using a 3-electrode configuration.

#### CA measurements

A symmetric Zn‖Zn cell was employed, in which both electrodes were zinc foils. For the anolyte, the concentration of Zn(OH)_4_^2−^ was 0.2 mol l^−1^, and that of NaOH was 2 mol l^−1^. For the anolyte with added LiCl, the concentrations of LiCl were 5.0, 10.0, and 15.5 wt%, respectively. The CA tests were conducted at an overpotential of −150 mV at 28 °C.

#### LSV and Tafel measurements

A zinc foil glassy carbon electrode was selected to serve as the working electrode, a saturated calomel electrode was picked as the reference electrode, and a platinum plate electrode was selected as the counter electrode. For the anolyte, the concentration of Zn(OH)_4_^2−^ was 0.2 mol l^−1^, and that of NaOH was 2 mol l^−1^. For the anolyte with added LiCl, the concentrations of LiCl were 5.0, 10.0, and 15.5 wt%, respectively. For LSV measurements, the anolyte was tested at a 1 mV s^−1^ scan rate at 28 °C. For Tafel measurements, the anolyte was tested at a 10 mV s^−1^ scan rate at 28 °C.

#### CV measurements

A glassy carbon electrode was selected to serve as the working electrode, a saturated calomel electrode was picked as the reference electrode, and a platinum plate electrode was selected as the counter electrode. For the anolyte, the concentration of Zn(OH)_4_^2−^ was 0.2 mol l^−1^, and that of NaOH was 2 mol l^−1^. For the catholyte, the concentration of K_4_[Fe(CN)_6_] was 0.5 mol l^−1^, and that of NaOH was 0.5 mol l^−1^. For the electrolyte with added LiCl, the concentration of LiCl was 15.5 wt%. The anolyte and catholyte were tested at different scan rates (10 to 30 mV s^−1^) at 0 and 28 °C.

#### EIS measurements

EIS was carried out with a glassy carbon electrode serving as the working electrode, alongside a saturated calomel electrode as the reference electrode and a platinum plate as the counter electrode. For the anolyte, the Zn(OH)_4_^2−^ concentration was 0.2 mol l^−1^, and that of NaOH was 2 mol l^−1^. For the catholyte, the concentration of K_4_[Fe(CN)_6_] was 0.5 mol l^−1^, and that of NaOH was 0.5 mol l^−1^. For the electrolyte with added LiCl, the concentrations of LiCl were 5.0, 10.0, and 15.5 wt%, respectively. The anolyte and catholyte were measured within the frequency range spanning 0.1 Hz to 100 kHz, with measurements taken at 0 and 28 °C.

### Assembly of the flow battery

The AZFFB experiments were conducted using a battery fixture with an active area of 4.5 × 3 cm^2^ (LSB-3, Wuhan Zhisheng New Energy Co., Ltd.) and a design without flow channels. All the charge–discharge experiments of the AZFFBs were conducted using graphite felts (thickness: 4.35 mm) as electrodes and Nafion 115 membranes as proton-exchange membranes. To increase the hydrophilicity of the graphite felts, they were heated in a muffle furnace at 500 °C for 2 h before the experiments. A peristaltic pump circulated the electrolyte between the storage tank and the battery fixture, with the electrolyte flow rate set at 50 ml min^−1^. The flow battery was subjected to charge and discharge experiments using the LAND battery test system (CT2001A, Wuhan, China). The NEWARE high–low temperature test chamber (MGDW-408-40, Shenzhen, China) was used to control the ambient temperature.

#### Electrolyte preparation

The anolyte was prepared from ZnO, NaOH, and deionized water, where the white powdery ZnO was dissolved in NaOH solution under magnetic stirring until complete dissolution. The catholyte was prepared from K_4_[Fe(CN)_6_], NaOH, and deionized water, with K_4_[Fe(CN)_6_] fully dissolved in the NaOH solution under magnetic stirring. For the LiCl-containing battery experiments, LiCl was introduced into both the anolyte and catholyte, followed by continuous magnetic stirring until complete dissolution. The concentrations of the redox-active species and NaOH were determined based on the volume of water at room temperature (28 °C), while the amount of LiCl added was calculated relative to the mass of water and was independent of temperature. Considering the marked variation of electrolyte volume with temperature and the nonconstancy of molar concentration at low temperatures, this study employed mass fraction, independent of temperature, to represent the additive content.

#### Testing conditions

For all AZFFB experiments, the Zn(OH)_4_^2−^ in the zinc-based electrolyte was 0.2 mol l^−1^, and the concentration of NaOH was 2 mol l^−1^. The K_4_[Fe(CN)_6_] concentration was 0.5 mol l^−1^, and the concentration of NaOH was 0.5 mol l^−1^. For the additive-containing battery experiments, the LiCl content was 15.5 wt%. The anolyte and catholyte volumes were fixed at 20 ml, with charging terminated upon reaching either 70% SOC in the anolyte or a cutoff voltage of 2.3 V, and discharging stopped at 0.1 V. A current density of 40 mA cm^−2^ was applied at 28 °C, while a current density of 20 mA cm^−2^ was used at −20 °C and under variable-temperature operation. The adopted SOC and corresponding areal capacity fall within the ranges widely reported for AZFFBs, ensuring reversible zinc plating/stripping and reliable performance evaluation [23,26,43,44,54,59,60].

## Data Availability

The data supporting this article are included as part of the Supplementary Materials.

## Supplementary Materials

Supplementary 1 Figs. S1 to S33 Tables S1 to S4 Notes S1 to S3
