# Supplementary material for: Synergistic Solvation Strategy for Low-Temperature Alkaline Zinc−Ferricyanide Flow Battery
Source: Research (Wash D C). 2026 Feb 2;9:1118. doi: 10.34133/research.1118 (PMC12862133; doi:10.34133/research.1118)
Supplement: Supplementary 1 — Figs. S1 to S33 Tables S1 to S4 Notes S1 to S3 [file research.1118.f1.docx]

Supporting Information

Title

Synergistic Solvation Strategy for Low-Temperature Alkaline Zinc–Ferricyanide Flow Battery

**Authors**

Yalu Xin^1^, Chen Li^1^, Wei Gao^1^*, Yongping Chen^1, 2^*

**Affiliations**

^1^Key Laboratory of Energy Thermal Conversion and Control of Ministry of Education, School of Energy and Environment, Southeast University, Nanjing, Jiangsu 210096, P. R. China.

^2^Jiangsu Key Laboratory of Micro and Nano Heat Fluid Flow Technology and Energy Application, School of Environmental Science and Engineering, Suzhou University of Science and Technology, Suzhou, Jiangsu 215009, P. R. China.

^*^Address correspondence to: Yongping Chen; ypchen@seu.edu.cn and Wei Gao; weigao@seu.edu.cn

Supplementary Notes (Note S1 to S3)

Supplementary Figures (Figure S1 to S33)

Supplementary Tables (Table S1 to S4)

References (1 to 24)

**Supplementary Notes**

**Note S1. Density functional theory (DFT) calculations of adsorption energy**

DFT is used to study the adsorption energy of several substances. All calculations are performed using ORCA (version 6.0.1)^1-4^ program at the B3LYP-D3^5, 6^ and def2-SVP^7^ basis set levels for the geometry optimization and vibration analysis. Single-point energy calculations are performed using the more accurate def2-TZVP^7^ basis set level. All calculations are performed under implicit aqueous solvents. The adsorption energy (*E*_ads_) is calculated using the following formula:

$\text{E}_{\text{ads}}\text{=}\text{E}_{\text{total}}\text{-}\text{E}_{\text{slab}}\text{-}\text{E}_{\text{adsorbate}}$ (1)

where *E*_total_, *E*_slab_, and *E*_adsorbate_ represent the total energy of the adsorption system, the energy of the slab, and the energy of the adsorbate, respectively.

Electrostatic potential (ESP) is calculated using Multiwfn (version 3.8)^8, 9^, and the software VMD^10^ is used for plotting.

**Note S2. Molecular dynamics (MD) simulations of electrolyte solvation structure**

Molecular dynamics (MD) simulations were performed on multiple systems to investigate their structural and dynamic properties. The composition of System 1 was as follows: Zn(OH)_4_^2-^ (8), Na⁺ (93), OH^-^ (77), Li⁺ (145), Cl^-^ (145), and H_2_O (2000). System 2 consisted of K⁺ (76), Fe(CN)_6_^4-^ (19), Na⁺ (19), OH^-^ (19), Li⁺ (145), Cl^-^ (145), and H_2_O (2000). System 3 included K⁺ (76), Fe(CN)_6_^4-^ (19), Na⁺ (19), OH^-^ (19), and H_2_O (2000). The initial configurations of these systems were generated using the PACKMOL package, with each simulation box having dimensions of 4 nm × 4 nm × 4 nm. All MD simulations were conducted using GROMACS 2023^11^. The General Amber Force Field (GAFF) was applied to describe the electrolyte system^12, 13^. Li et al. optimized the ion force field used for these metal ions better to describe the solvation layer structure of metal ions^14^. The OPC3 model was used for water molecules. Long-range electrostatic interactions were computed using the particle-mesh Ewald (PME) method, with a real-space cutoff of 1.0 nm^15^. Van der Waals interactions were truncated at the same cutoff distance of 1.0 nm. Prior to production runs, energy minimization was performed to ensure system stability. Equilibration was carried out in two stages: initially, the system was equilibrated for 1 ns at 300 K and 1 bar using a velocity-rescaling (V-rescale) thermostat and a Berendsen barostat. This was followed by an additional 1 ns equilibration in the isothermal-isobaric (NPT) ensemble at 300 K and 1 bar. After the annealing and equilibration phases, a 10 ns production MD simulation was conducted with a time step of 1 fs. Trajectories were recorded every 5000 ps under constant temperature (300 K) and pressure (1 bar) using a V-rescale thermostat. Trajectory visualization and analysis were performed using VMD 1.9.3^10^.

**Note S3. Calculation methods**

The migration rate of redox-active substances in the electrolyte is used to quantify the ionic diffusion coefficient (*D*), and the *D* of catholyte and anolyte are calculated by the Randles-Sevcik equation:

$\text{ }\text{D}\text{=}\left( \frac{\text{k}}{\text{2.69×}\text{10}^{\text{5}}\text{n}^{\text{3/2}}\text{AC}} \right)^{\text{2}}$ (2)

in which *k* represents the slope of the peak current of the CV curve under different sweep rates. *n* represents the number of electrons transferred in the redox process, *A* is the active area of the working electrode, and *C* is the concentration of the redox active substance.

Battery capacity (*Q*) and state of charge (SOC) are calculated by the following equations:

$\text{Q}\text{=}\frac{nCF}{3600}$ (4)

$\text{SOC}\text{=}\frac{\text{Q}_{\text{a}}}{\text{Q}_{\text{t}}}\text{×100\%}$ (5)

where *n* represents the number of electrons transferred in electrolytic reactions, *C* is the concentration of the electrolyte, and its upper limit is the solubility of the electrolyte. *F* is the Faraday constant (~96485 C mol^-1^). *Q*_a_ and *Q*_t_ refers to the actual capacity and theoretical capacity, respectively.

The efficiency of the flow battery was evaluated through Coulomb efficiency (CE) and energy efficiency (EE). The calculation formula for CE and EE are as follows:

$\text{CE=}\frac{\int\text{I}_{\text{d}}\text{dt}}{\int\text{I}_{\text{c}}\text{dt}}\text{×100\%}$ (6)

$\text{E}\text{E=}\frac{\int V_{d}\text{I}_{\text{d}}\text{dt}}{\int V_{c}\text{I}_{\text{c}}\text{dt}}\text{×100\%}$ (7)

in which *I*_c_ and *I*_d_ refers to the battery charge/discharge current value, respectively.

**Supplementary Figures**


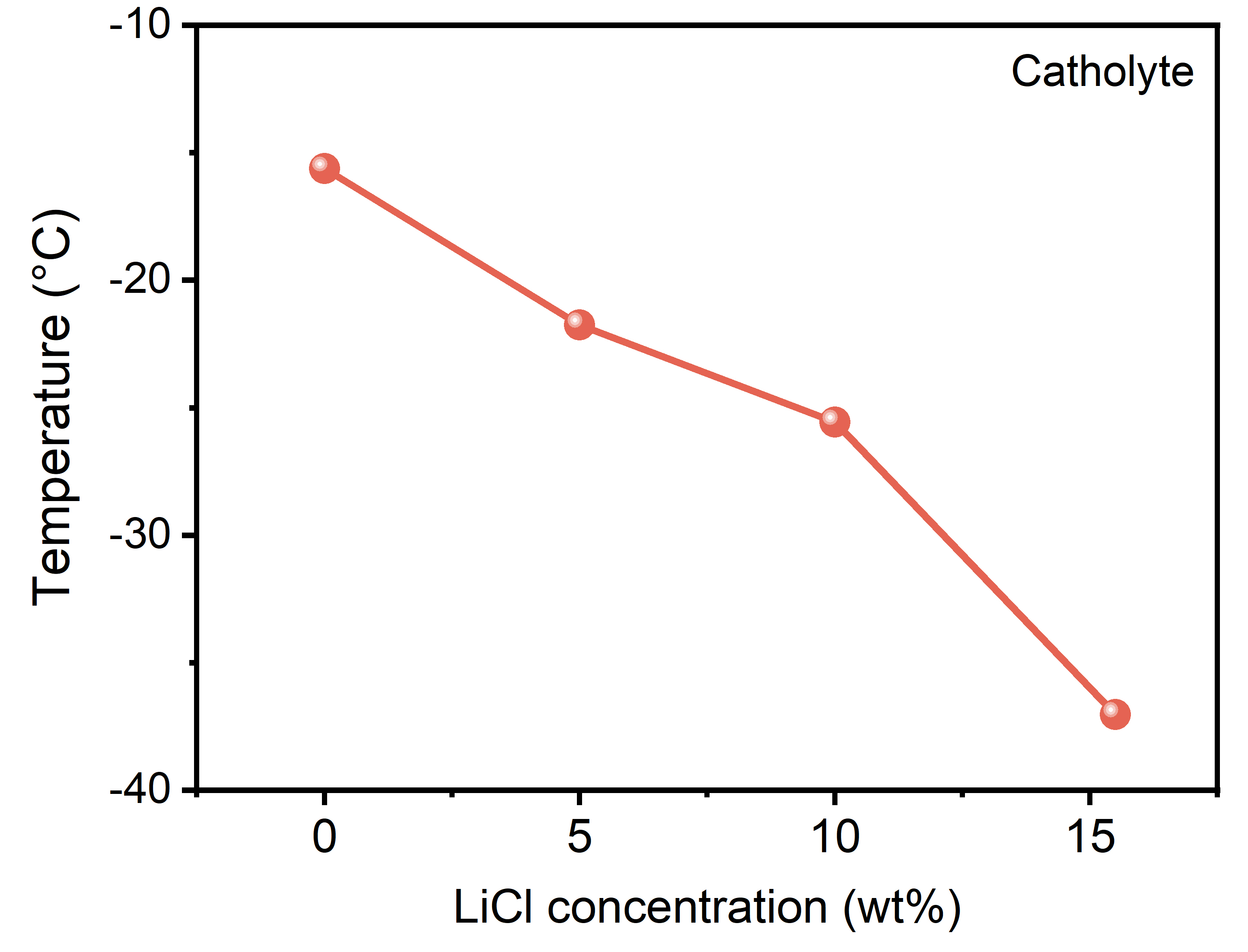


**Figure S1.** *T*_t_ values of the catholyte vary with the concentration of LiCl.


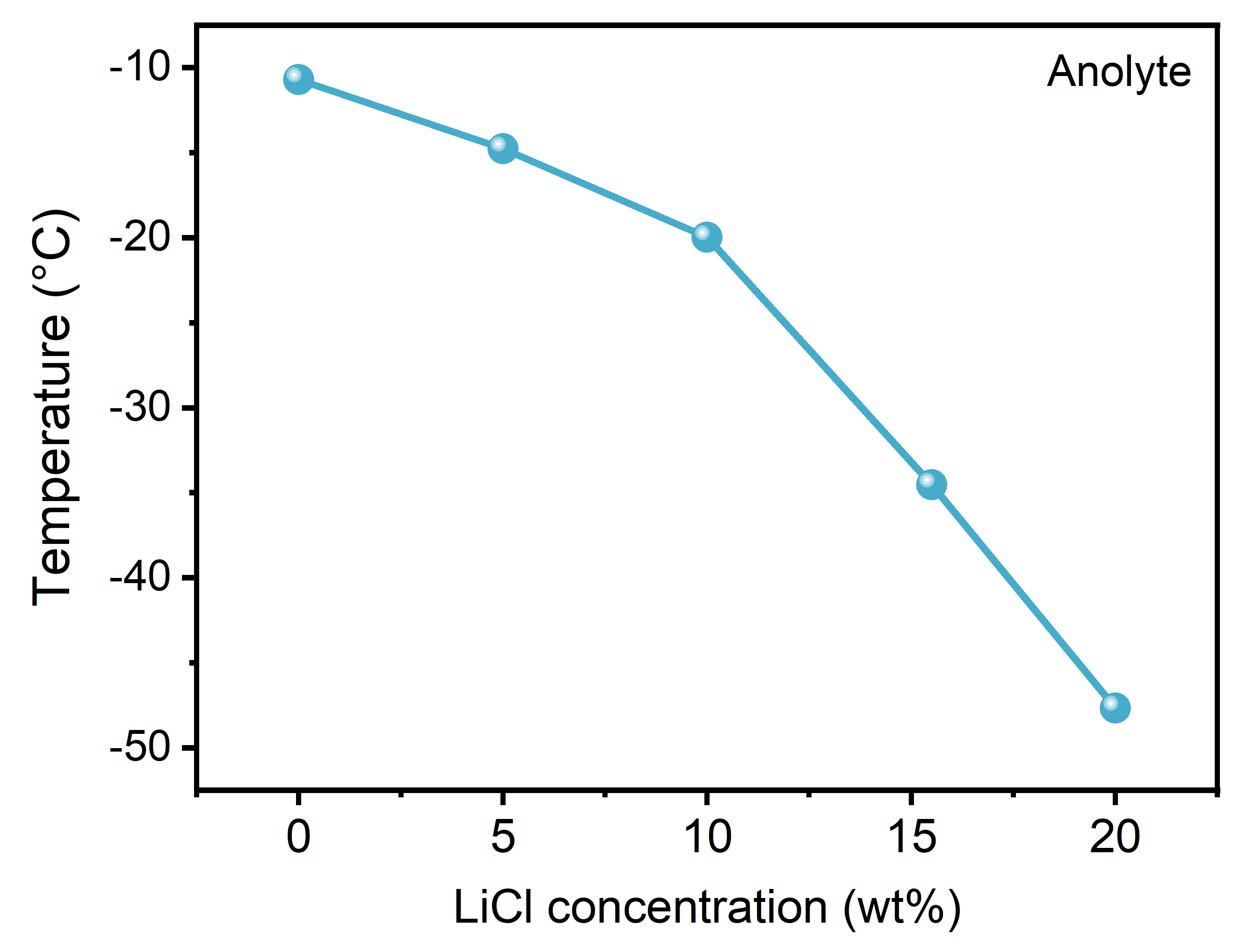


**Figure S2.** *T*_t_ values of the anolyte vary with the concentration of LiCl.


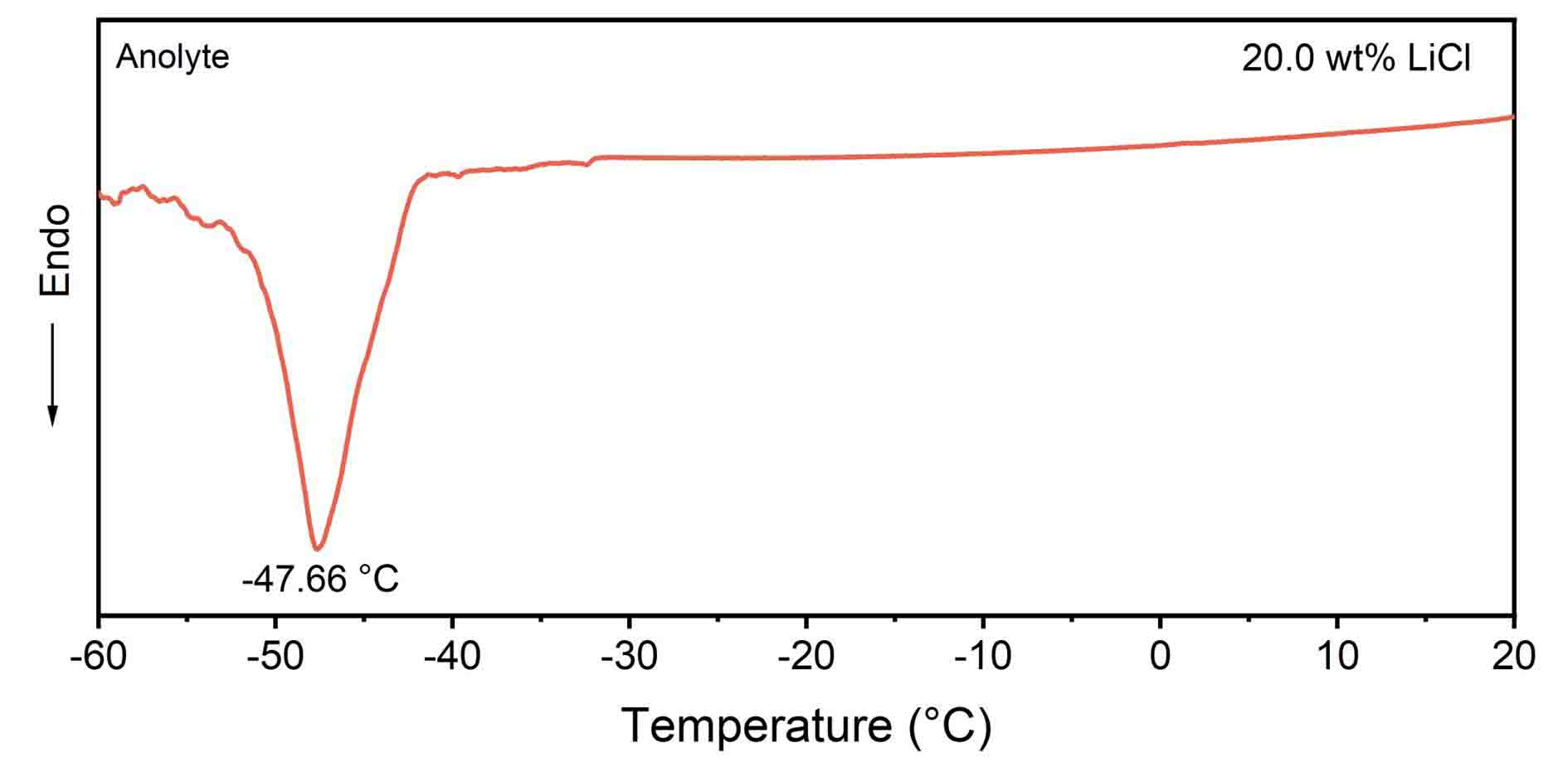


**Figure S3.** Anolyte’s *T*_t_ values with the 20.0 wt% concentration of LiCl.


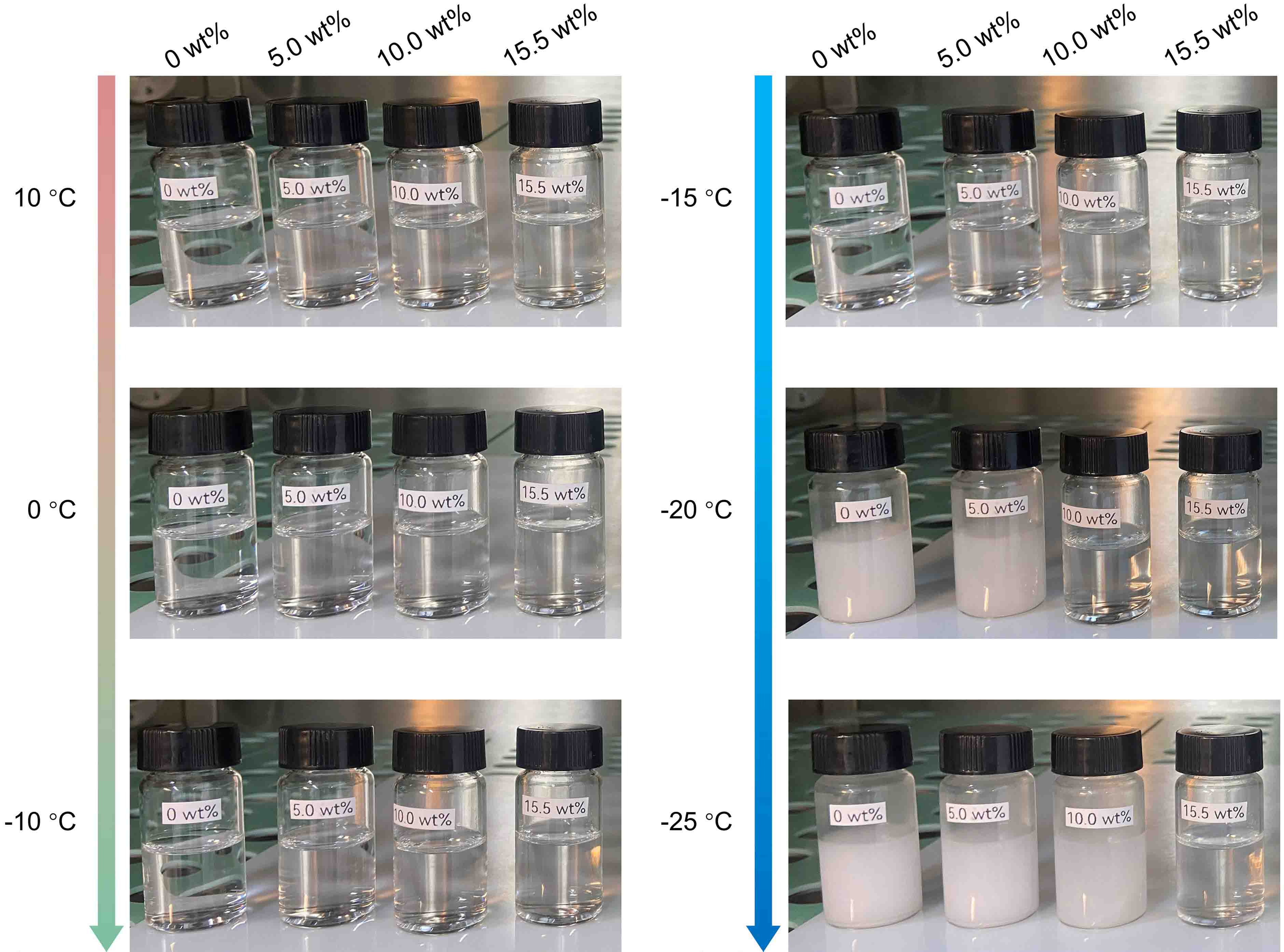


**Figure S4.** Optical images of anolyte with and without Li^+^ and Cl^-^ at different ambient temperatures.


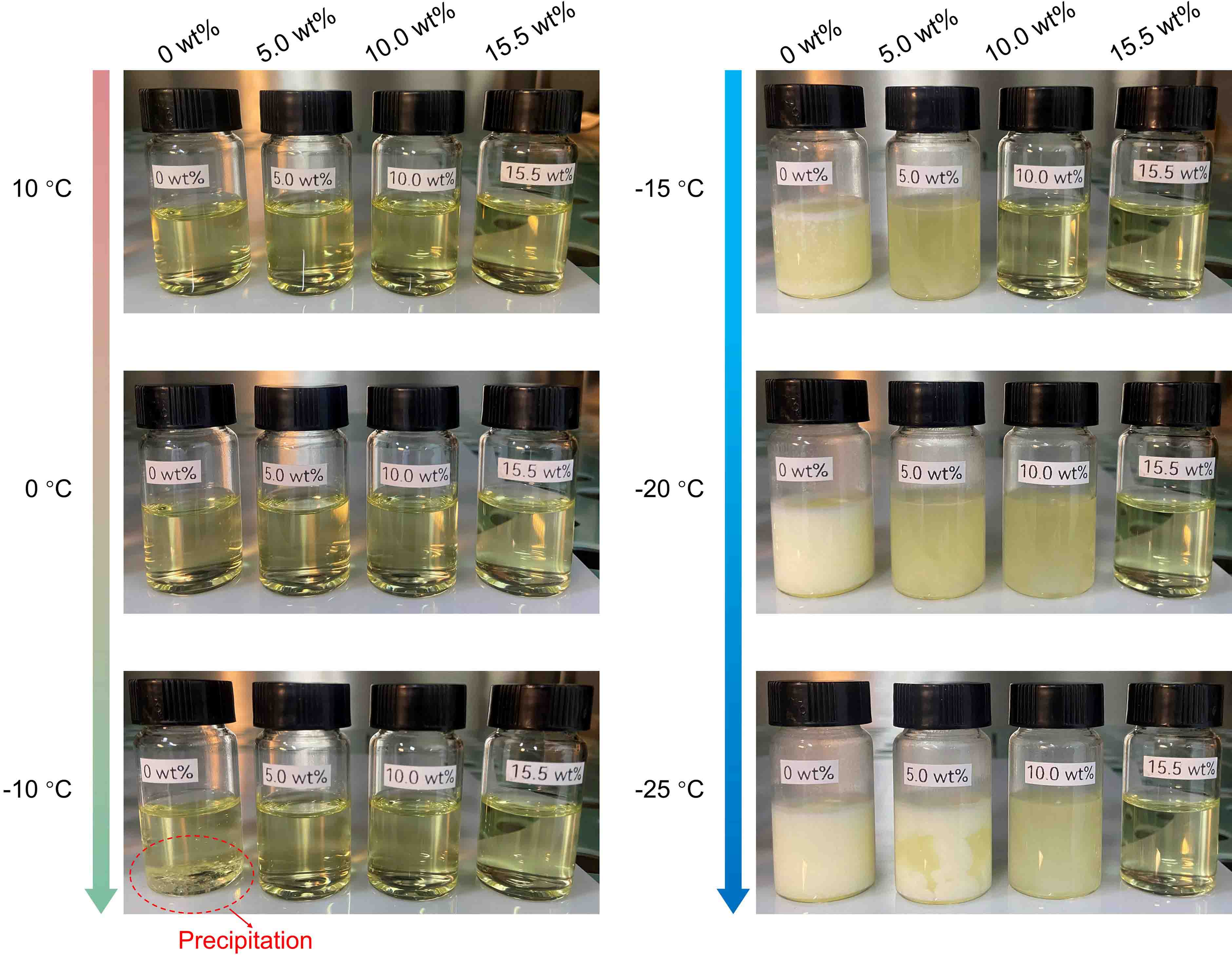


**Figure S5.** Optical images of catholyte with and without Li^+^ and Cl^-^ at different ambient temperatures.


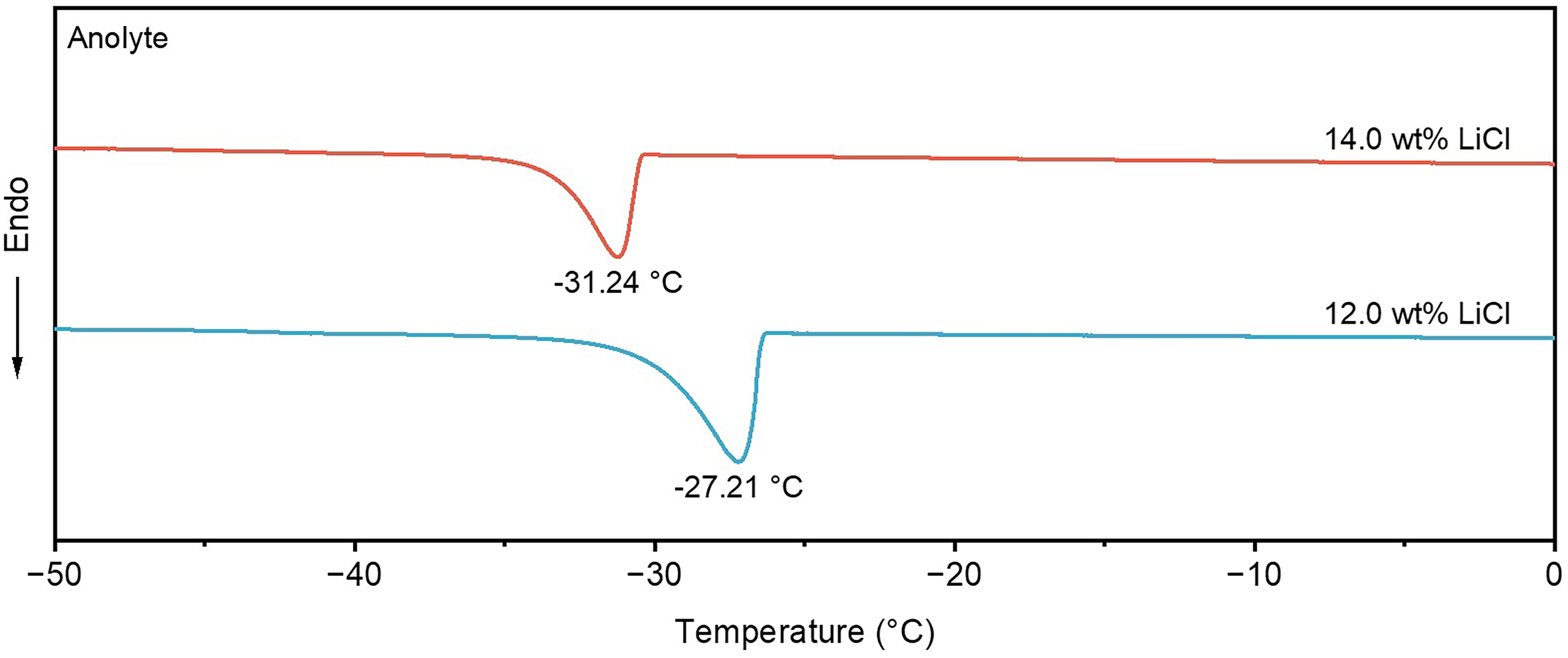


**Figure S6.** Anolyte’s *T*_t_ values with 12.0 wt% and 14.0 wt% concentrations of LiCl.


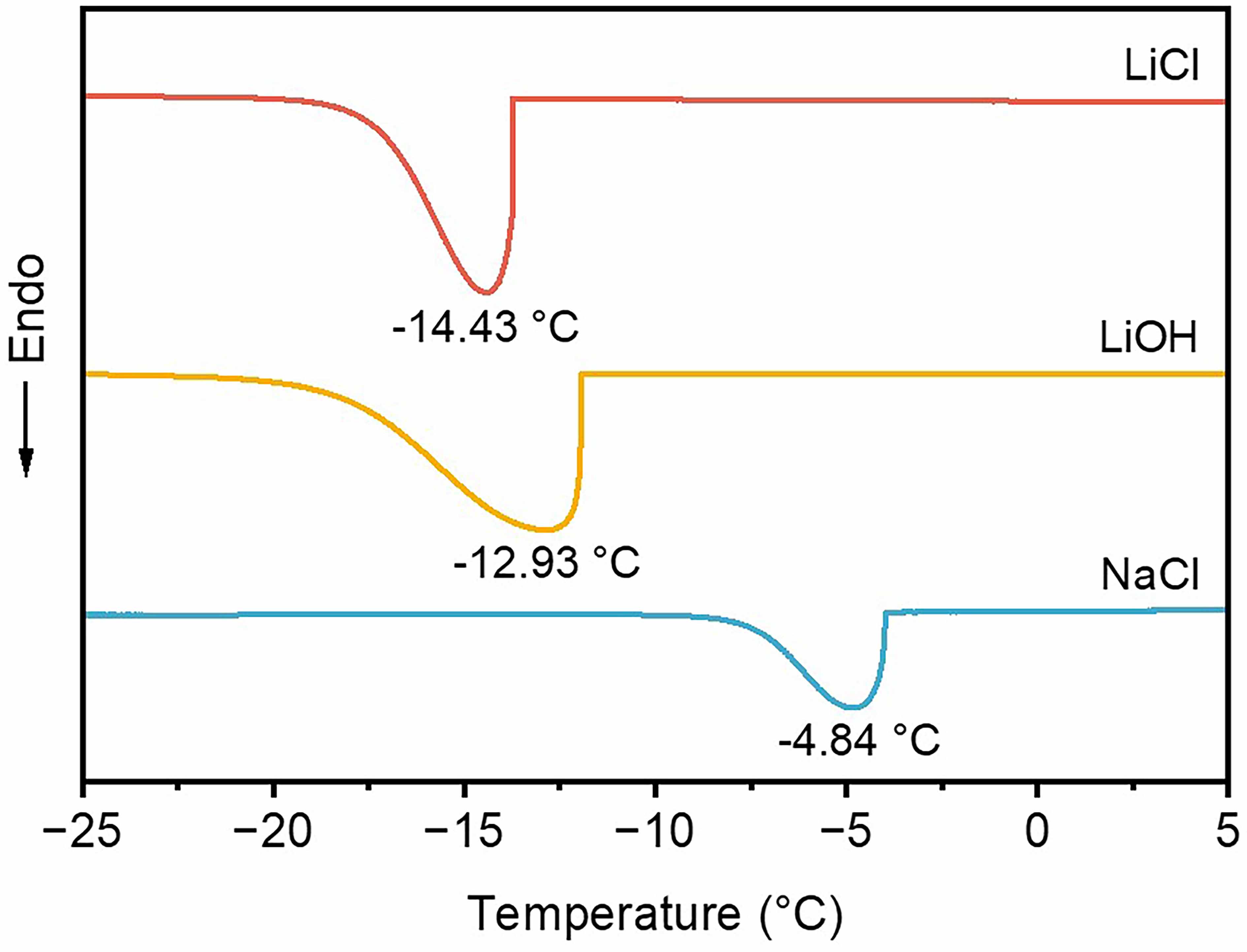


**Figure S7.** *T*_t_ values of 1 M NaCl, 1 M LiOH, and 1 M LiCl aqueous solutions.


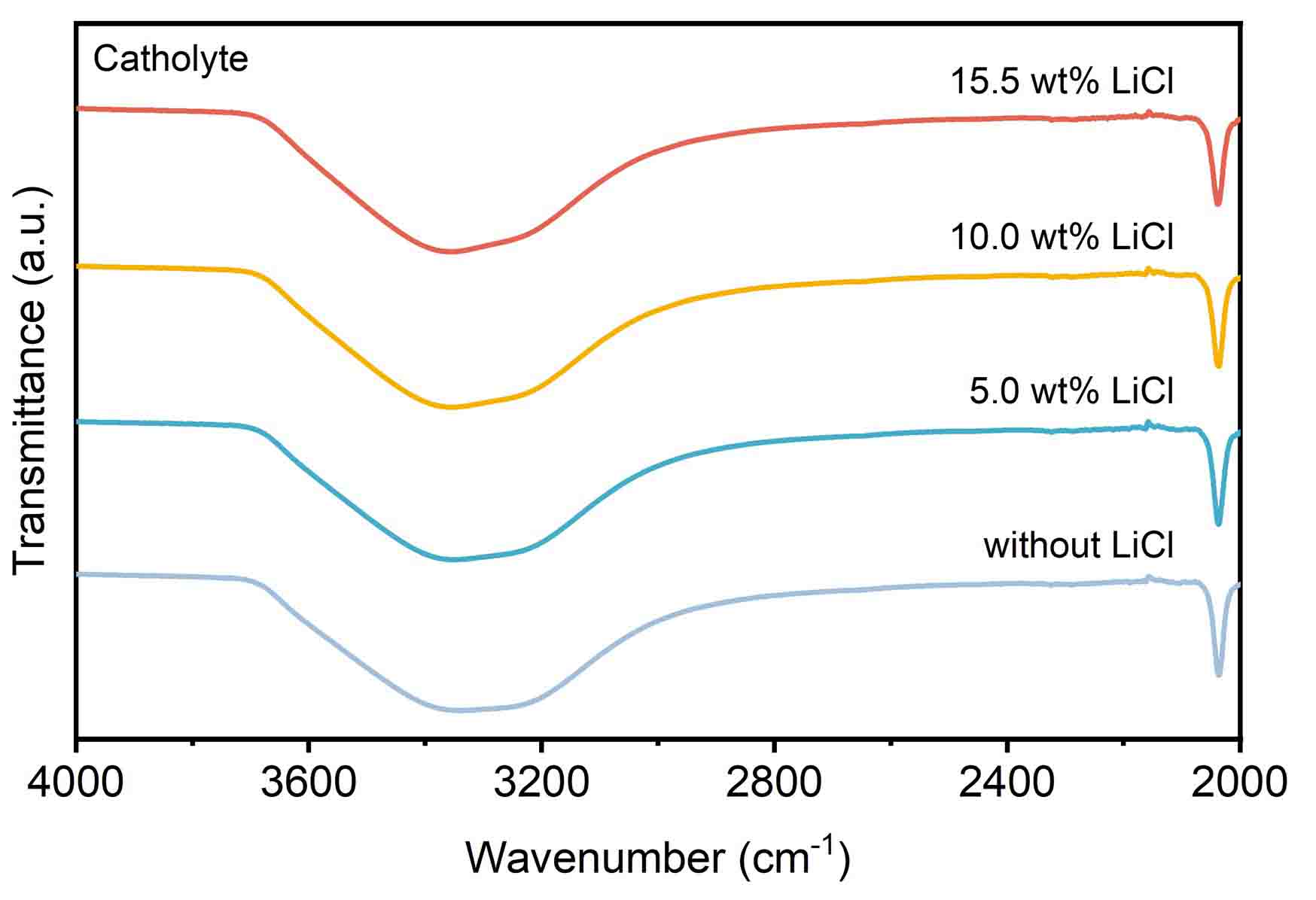


**Figure S8.** FTIR spectra of catholyte in 4000 ~ 2000 cm^-^¹ at room temperature.


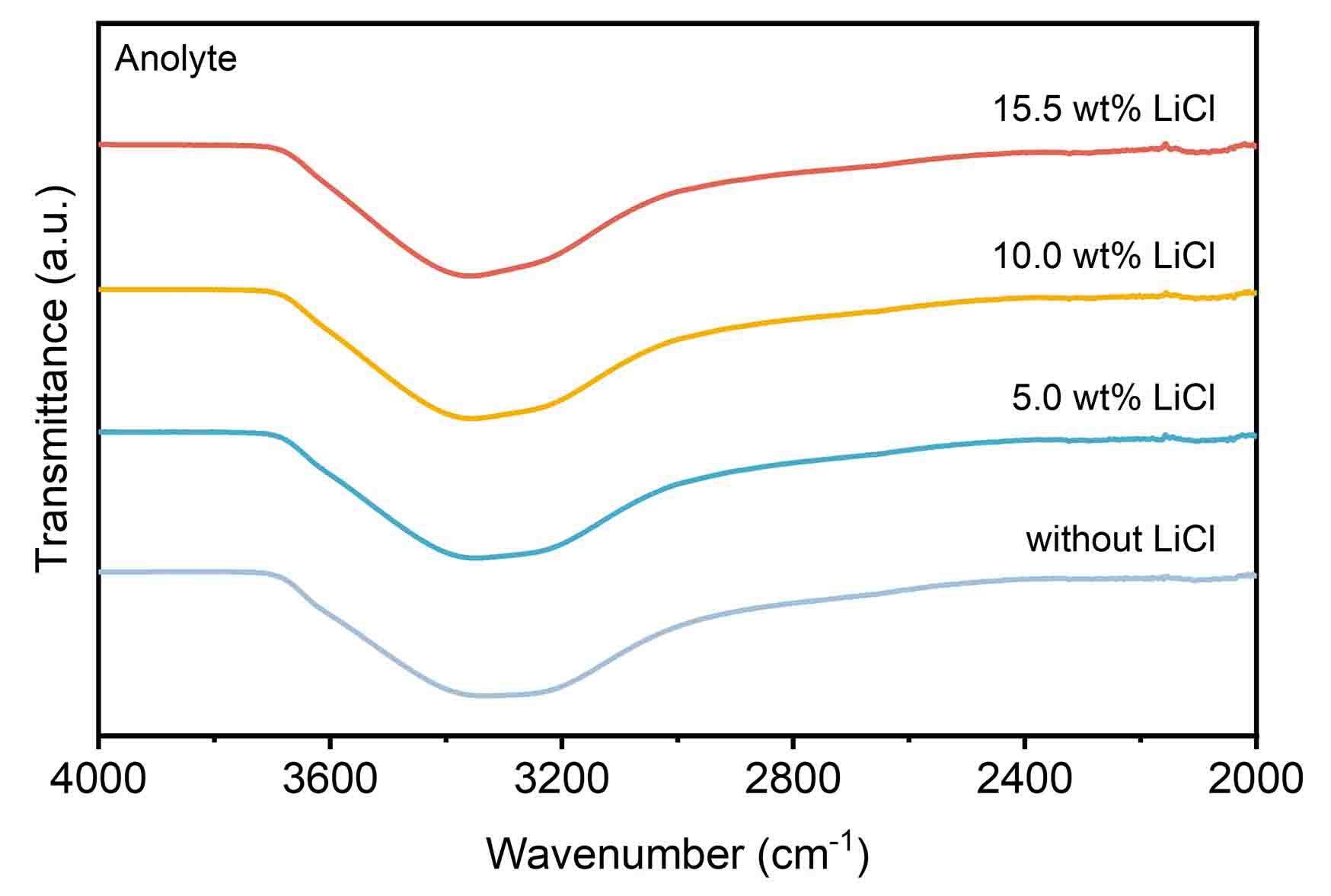


**Figure S9.** FTIR spectra of anolyte in 4000 ~ 2000 cm^-^¹ at room temperature.


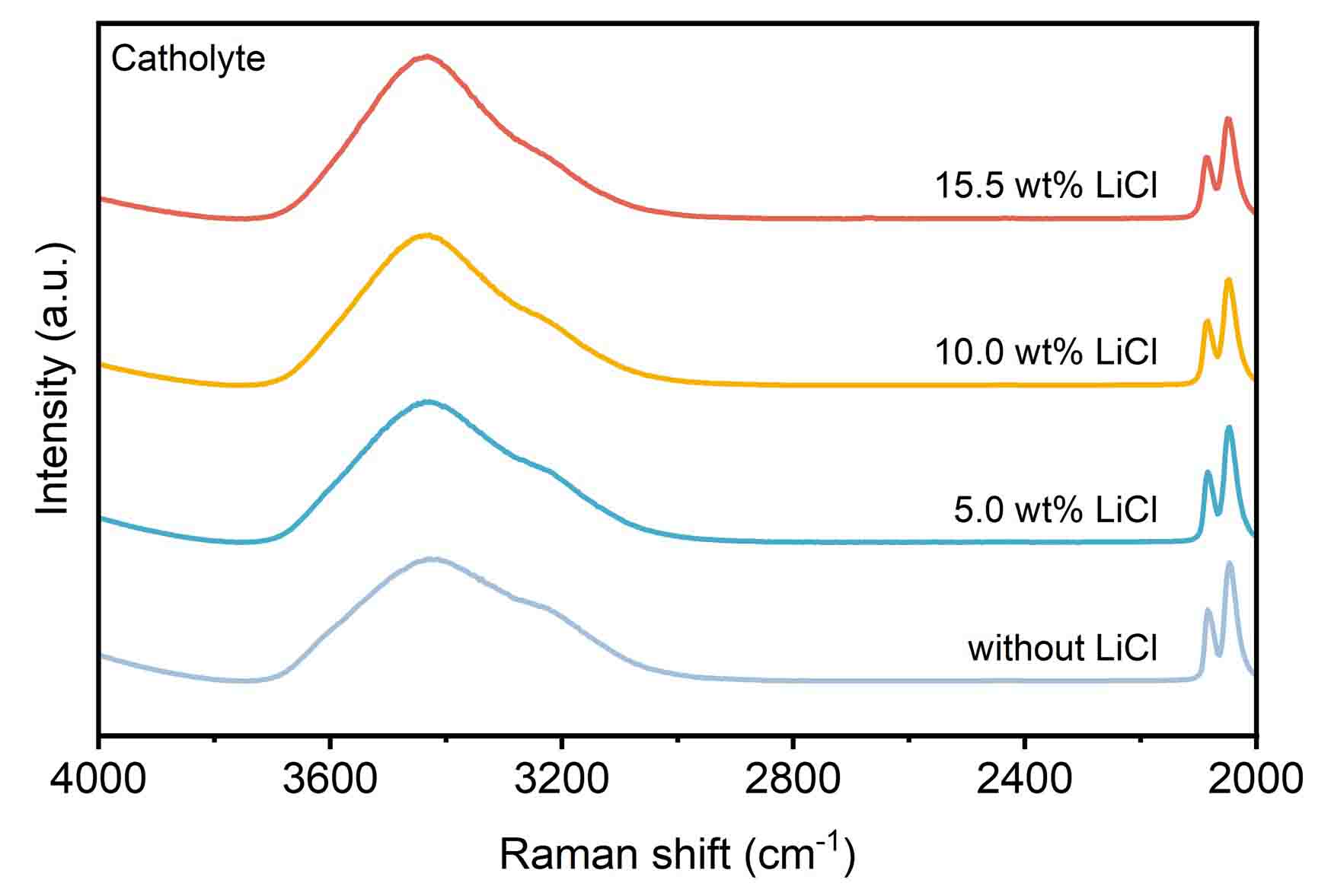


**Figure S10.** Raman spectra of catholyte in 4000 ~ 2000 cm^-^¹ at room temperature.


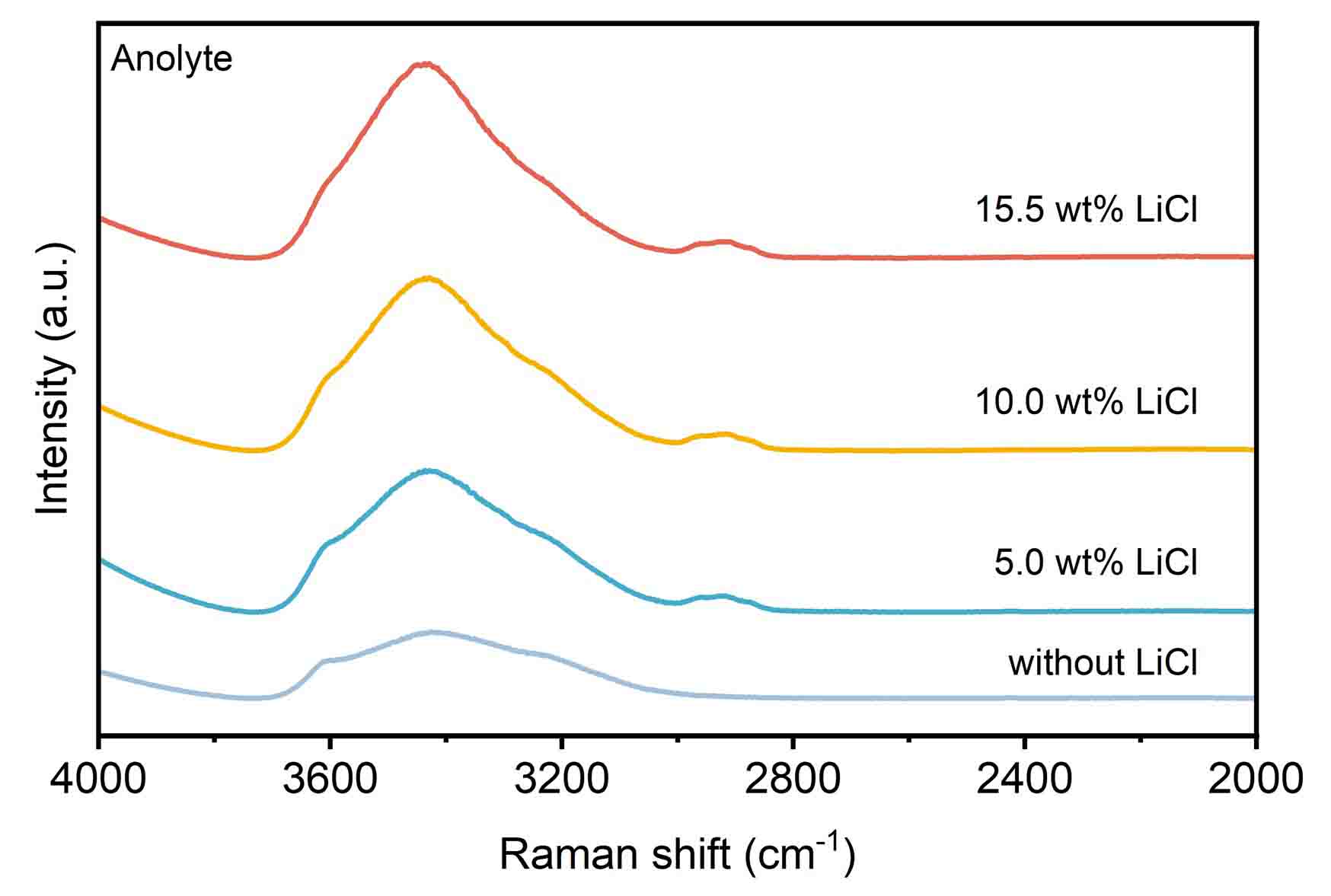


**Figure S11.** Raman spectra of anolyte in 4000 ~ 2000 cm^-^¹ at room temperature.

**
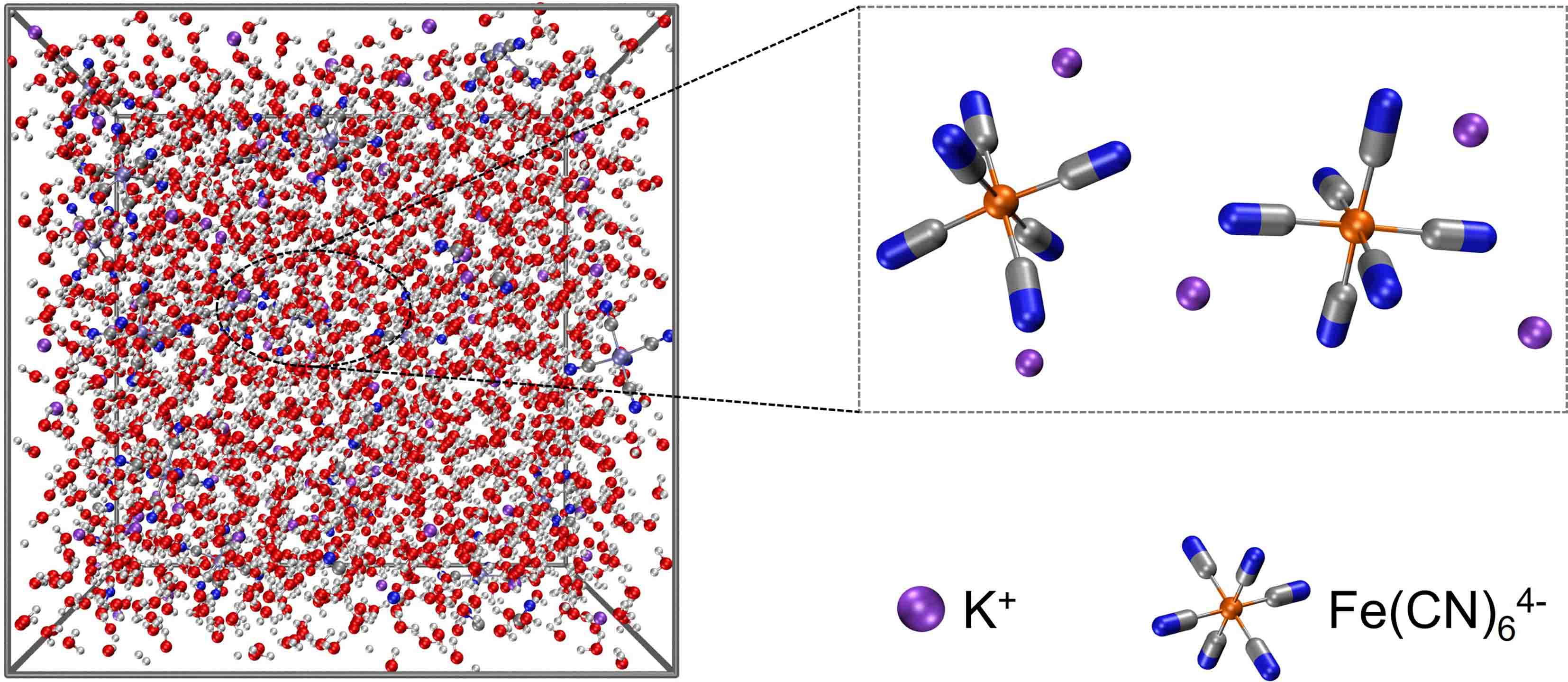
**

**Figure S12.** MD simulation of catholyte without Li^+^ and Cl^-^.

**
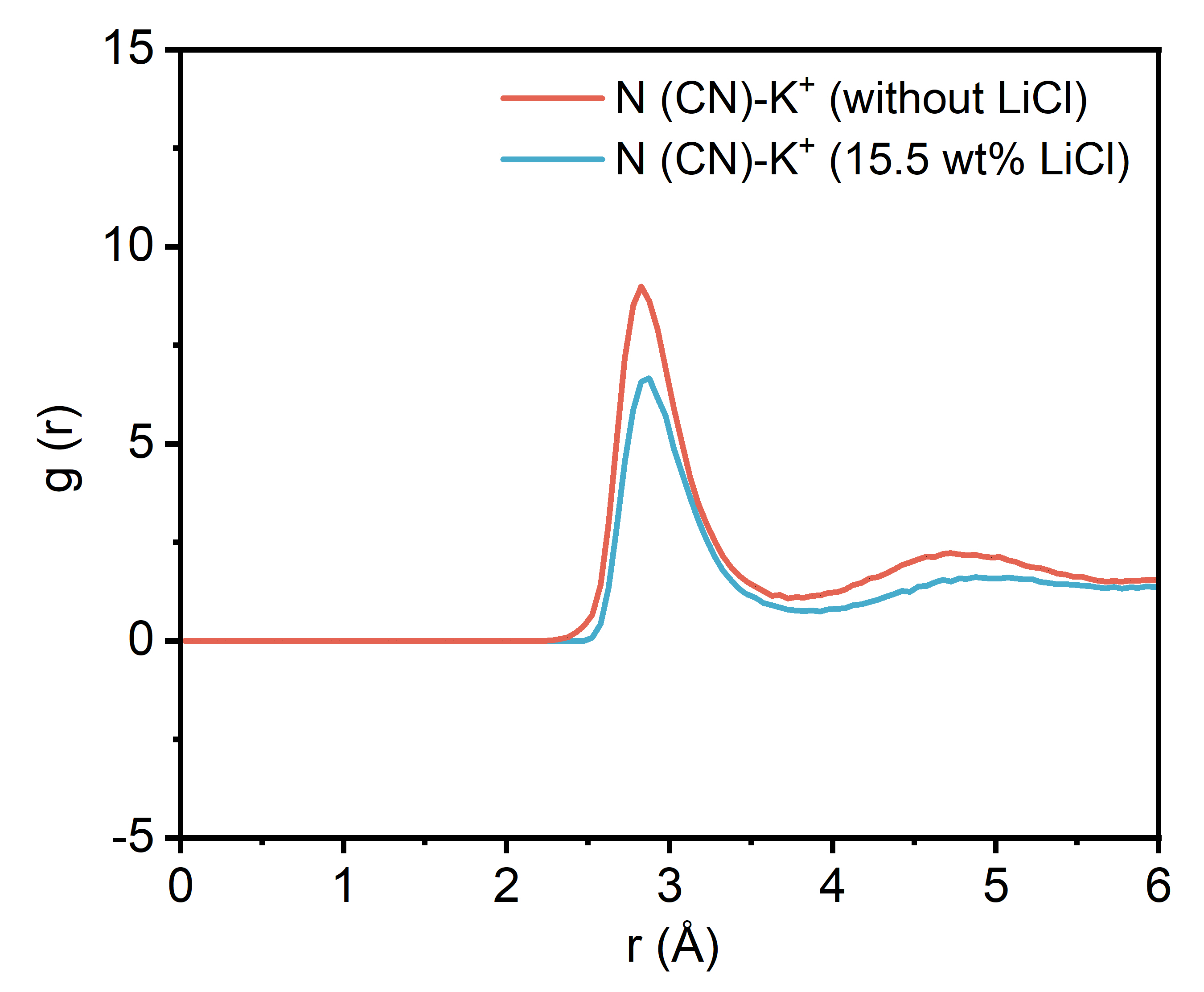
**

**Figure S13.** Radial distribution functions of K^+^ in catholyte with and without Li^+^ and Cl^-^.


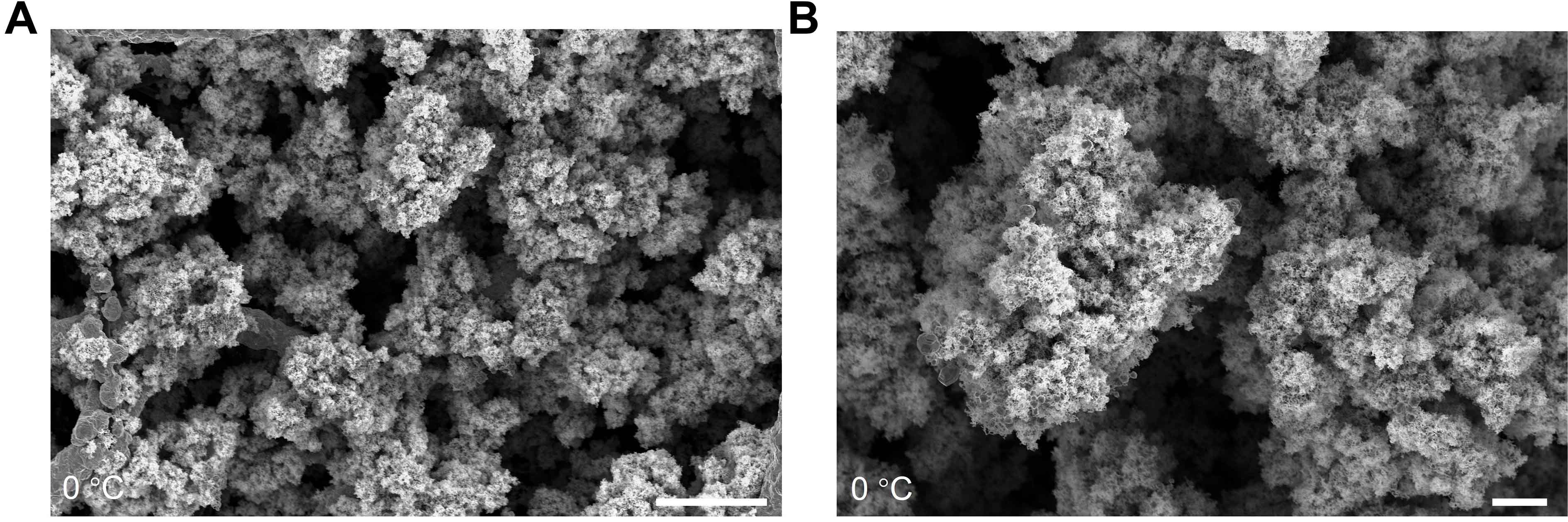


**Figure S14.** SEM images of zinc deposited on the surface of the graphite felt electrode at 10 mAh cm^-2^ areal capacity and 10 mA cm^-2^ current density in the anolyte without LiCl at 0 °C. (A) Scale bar: 100 μm. (B) Scale bar: 20 μm.


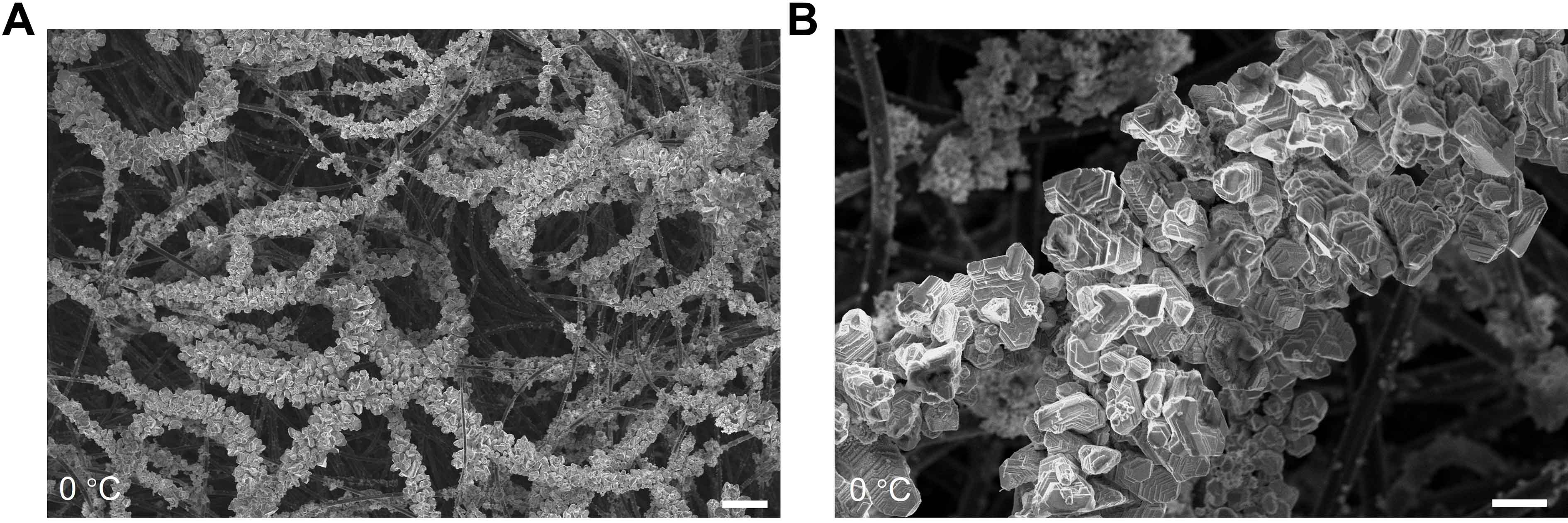


**Figure S15.** SEM images of zinc deposited on the surface of the graphite felt electrode at 10 mAh cm^-2^ areal capacity and 10 mA cm^-2^ current density in the anolyte with LiCl at 0 °C. (A) Scale bar: 100 μm. (B) Scale bar: 20 μm.


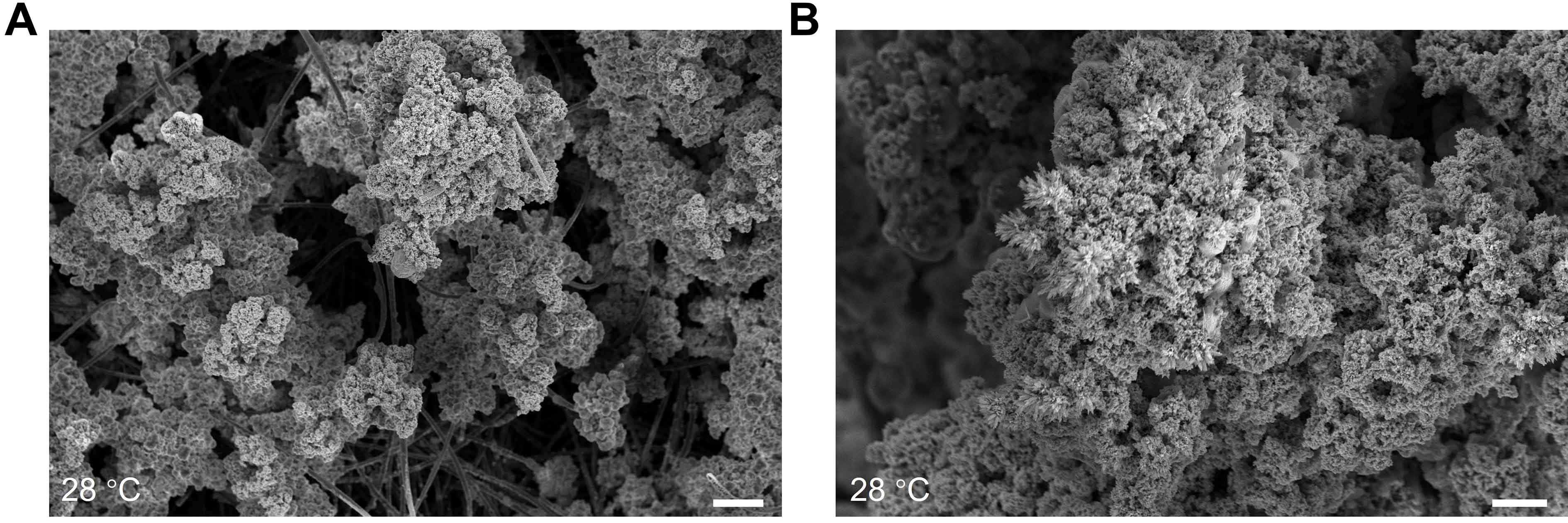


**Figure S16.** SEM images of zinc deposited on the surface of the graphite felt electrode at 10 mAh cm^-2^ areal capacity and 10 mA cm^-2^ current density in the anolyte without LiCl at 28 °C. (A) Scale bar: 100 μm. (B) Scale bar: 20 μm.


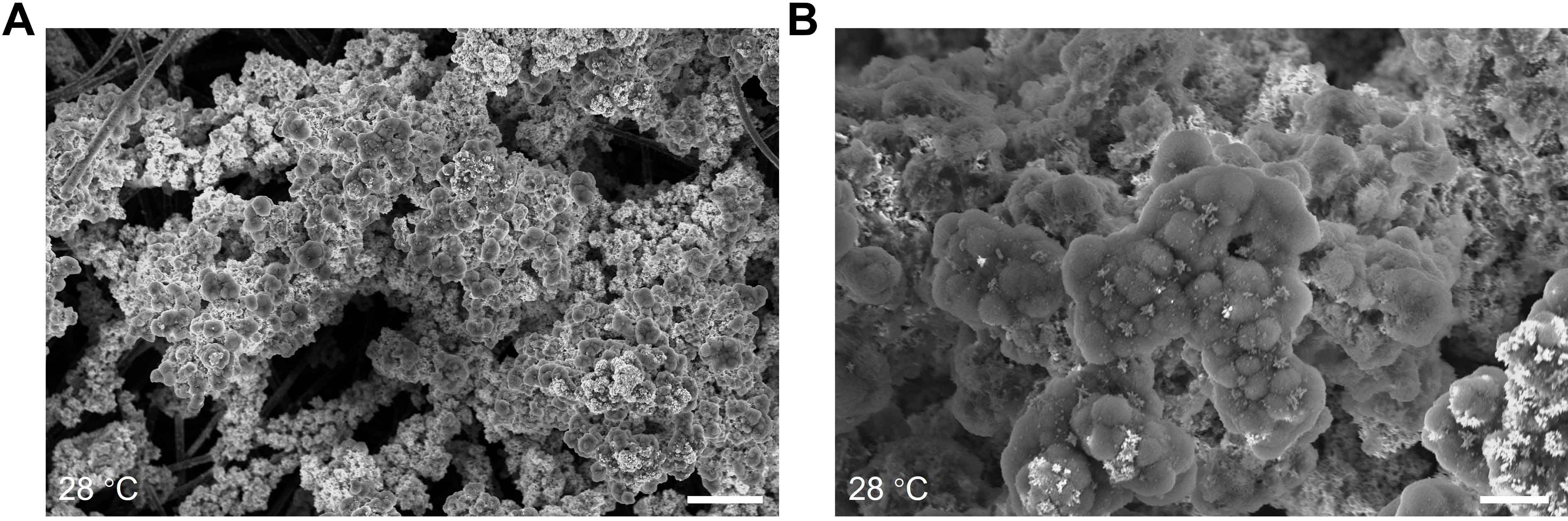


**Figure S17.** SEM images of zinc deposited on the surface of the graphite felt electrode at 10 mAh cm^-2^ areal capacity and 10 mA cm^-2^ current density in the anolyte with LiCl at 28 °C. (A) Scale bar: 100 μm. (B) Scale bar: 20 μm.


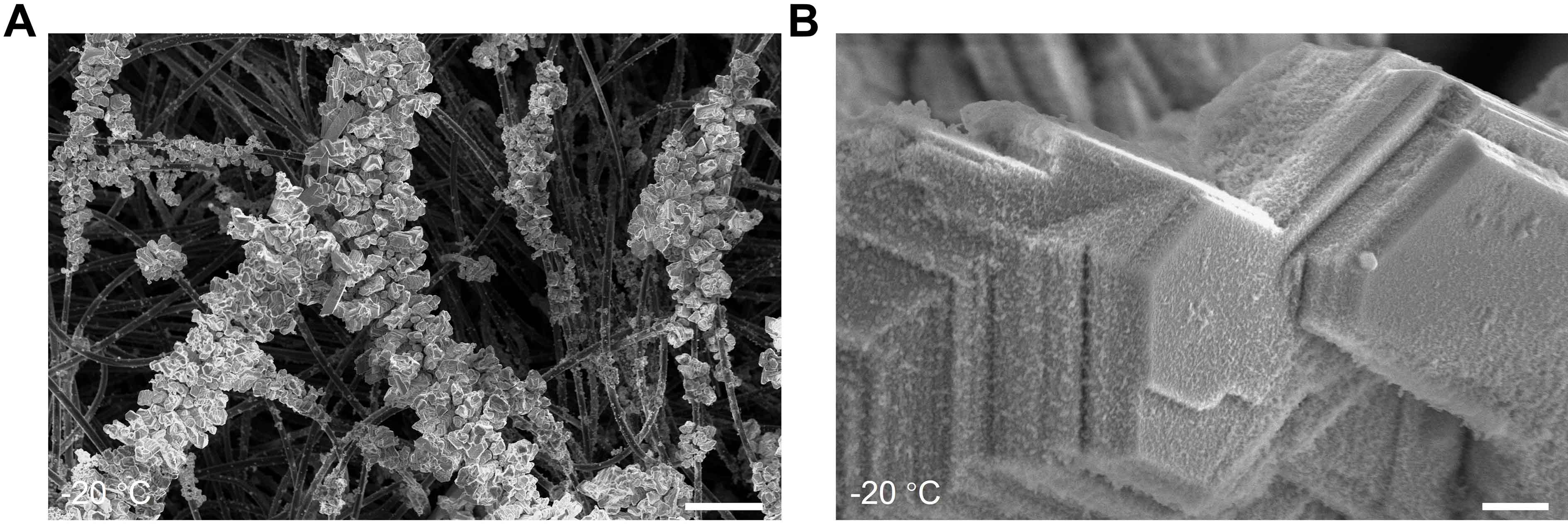


**Figure S18.** SEM images of zinc deposited on the surface of the graphite felt electrode at 10 mAh cm^-2^ areal capacity and 10 mA cm^-2^ current density in the anolyte with LiCl at -20 °C. (A) Scale bar: 100 μm. (B) Scale bar: 2 μm.


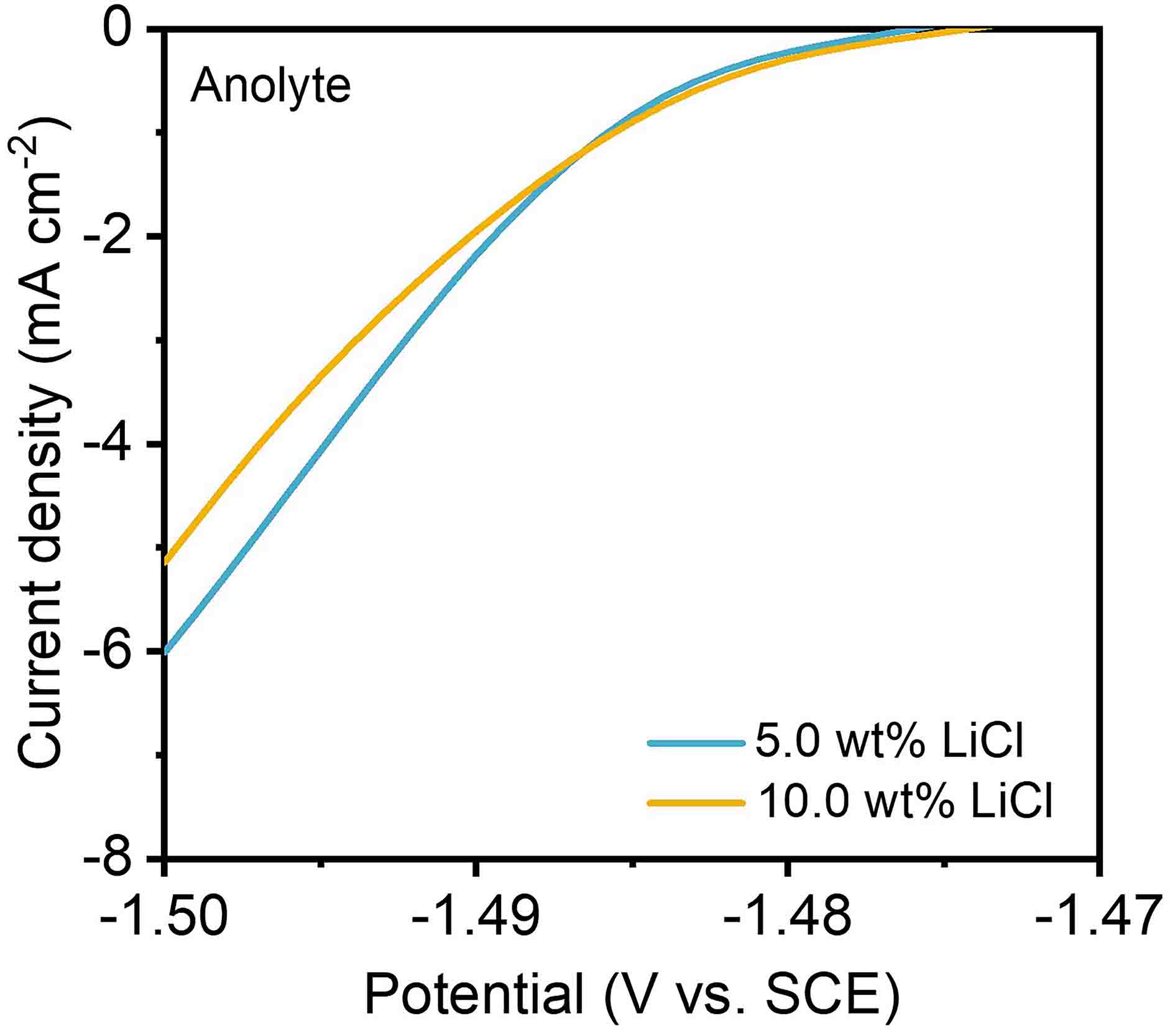


**Figure S19.** LSV curves of the anolyte with 5.0 wt% and 10.0 wt% LiCl.


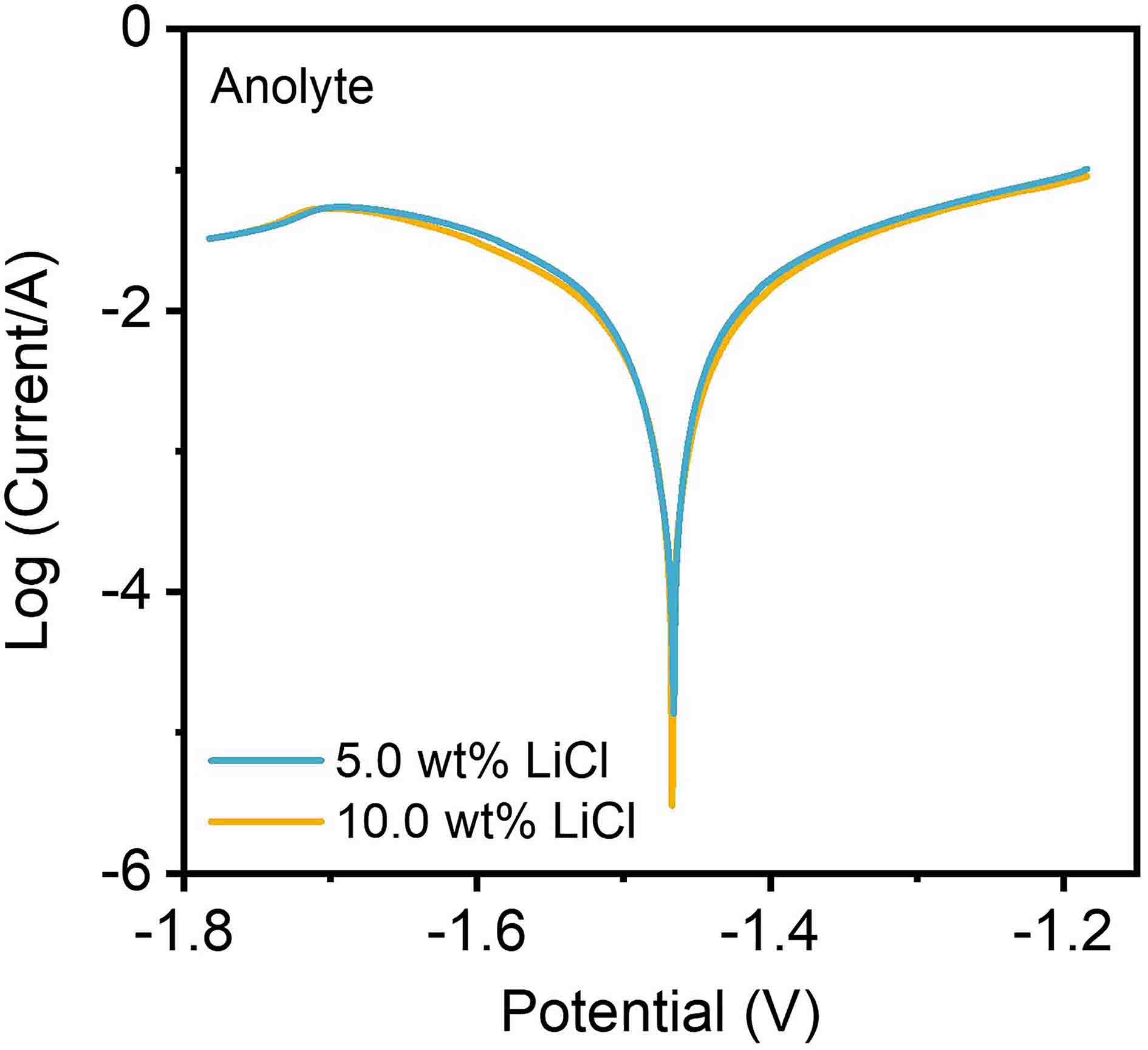


**Figure S20.** Tafel curves of the anolyte with 5.0 wt% and 10.0 wt% LiCl.


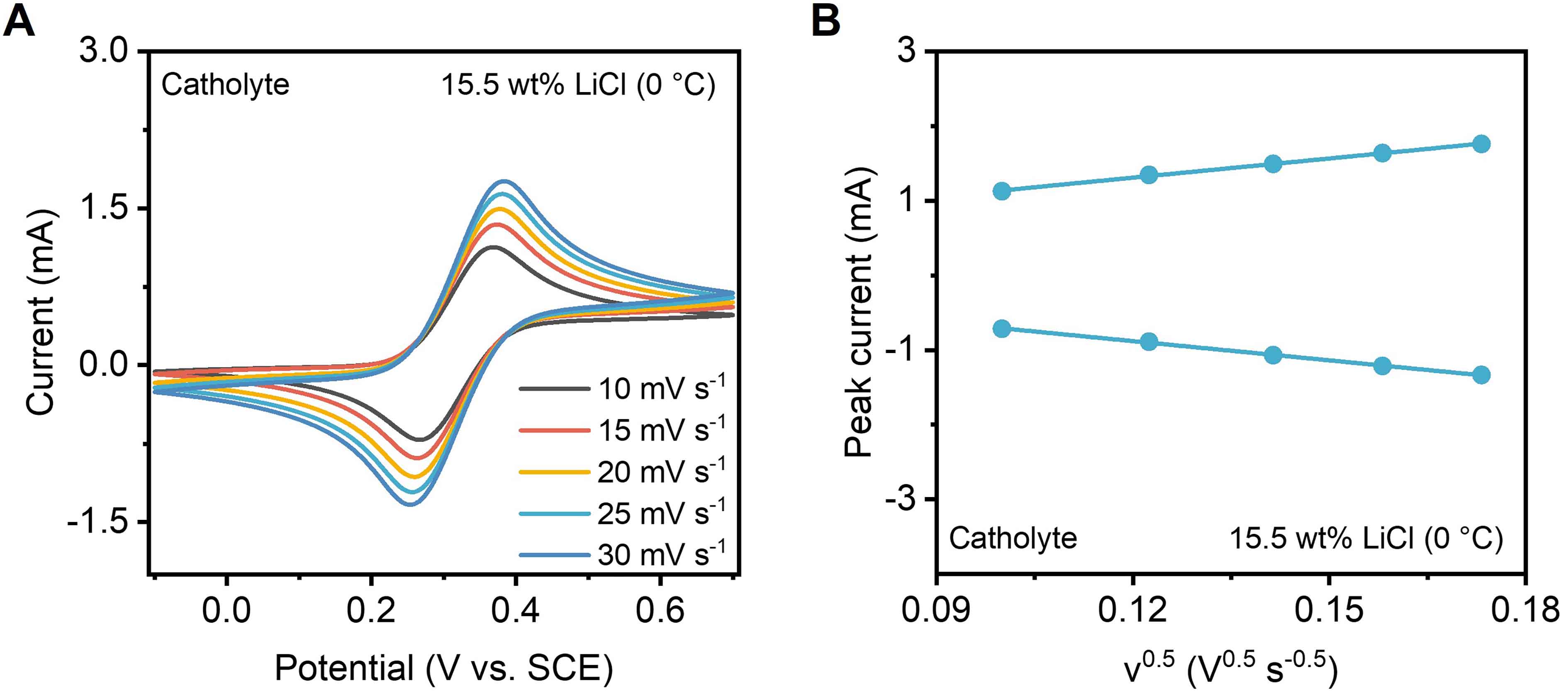


**Figure S21.** Electrochemical kinetics and diffusion-controlled behavior of the catholyte with 15.5 wt% LiCl at 0 °C. (A) CV curves at different scan rates. (B) The linear relationship between the oxidation and reduction peak currents and the square root of the scan rate.


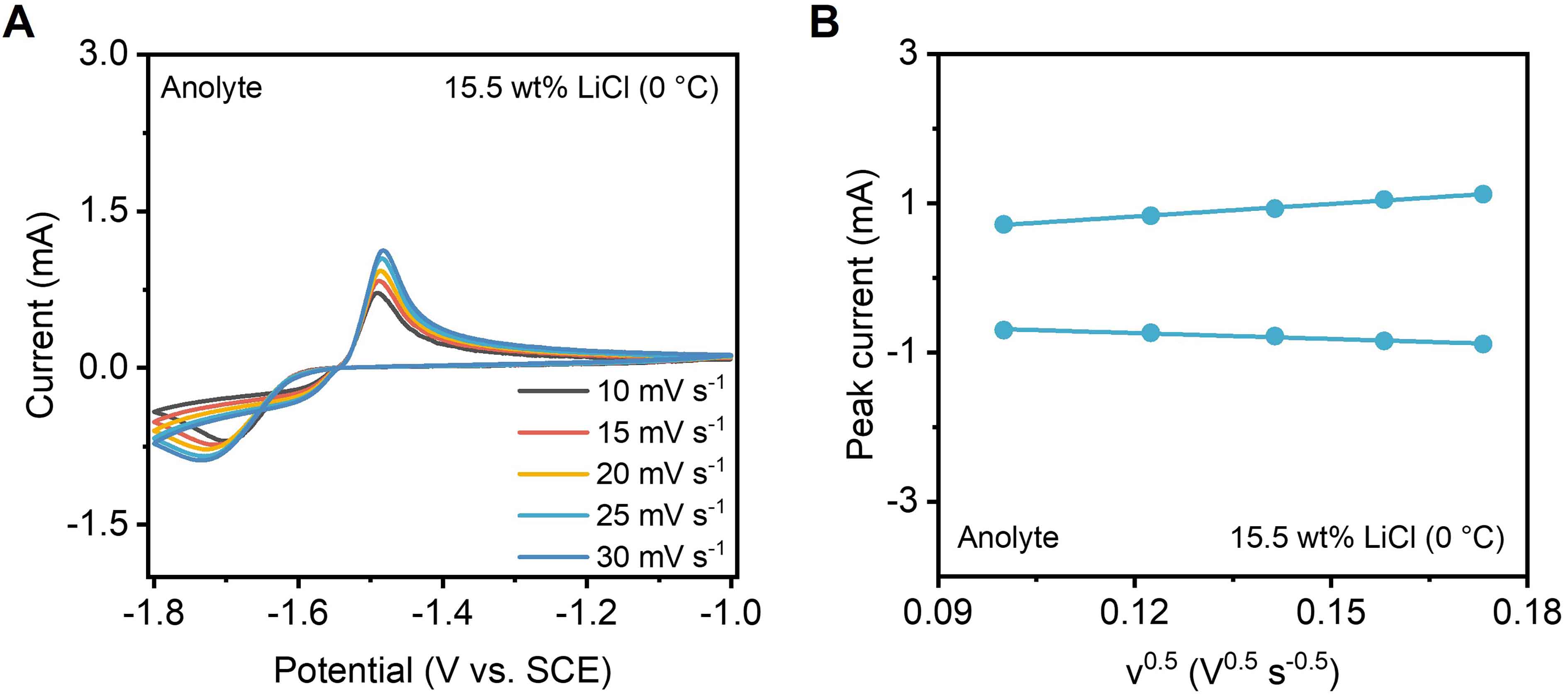


**Figure S22.** Electrochemical kinetics and diffusion-controlled behavior of the anolyte with 15.5 wt% LiCl at 0 °C. (A) CV curves at different scan rates. (B) The linear relationship between the oxidation and reduction peak currents and the square root of the scan rate.


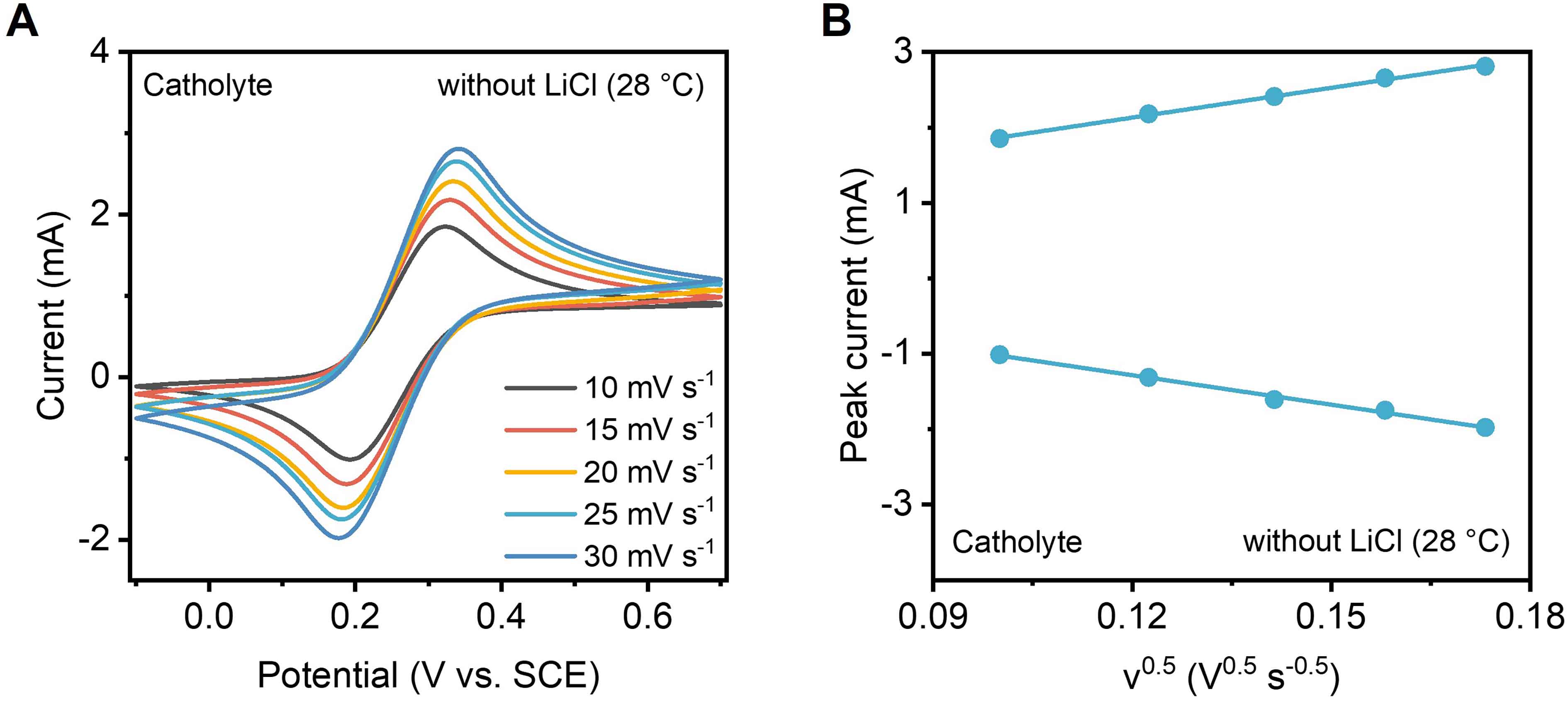


**Figure S23.** Electrochemical kinetics and diffusion-controlled behavior of the LiCl-free catholyte at 28 °C. (A) CV curves at different scan rates. (B) The linear relationship between the oxidation and reduction peak currents and the square root of the scan rate.


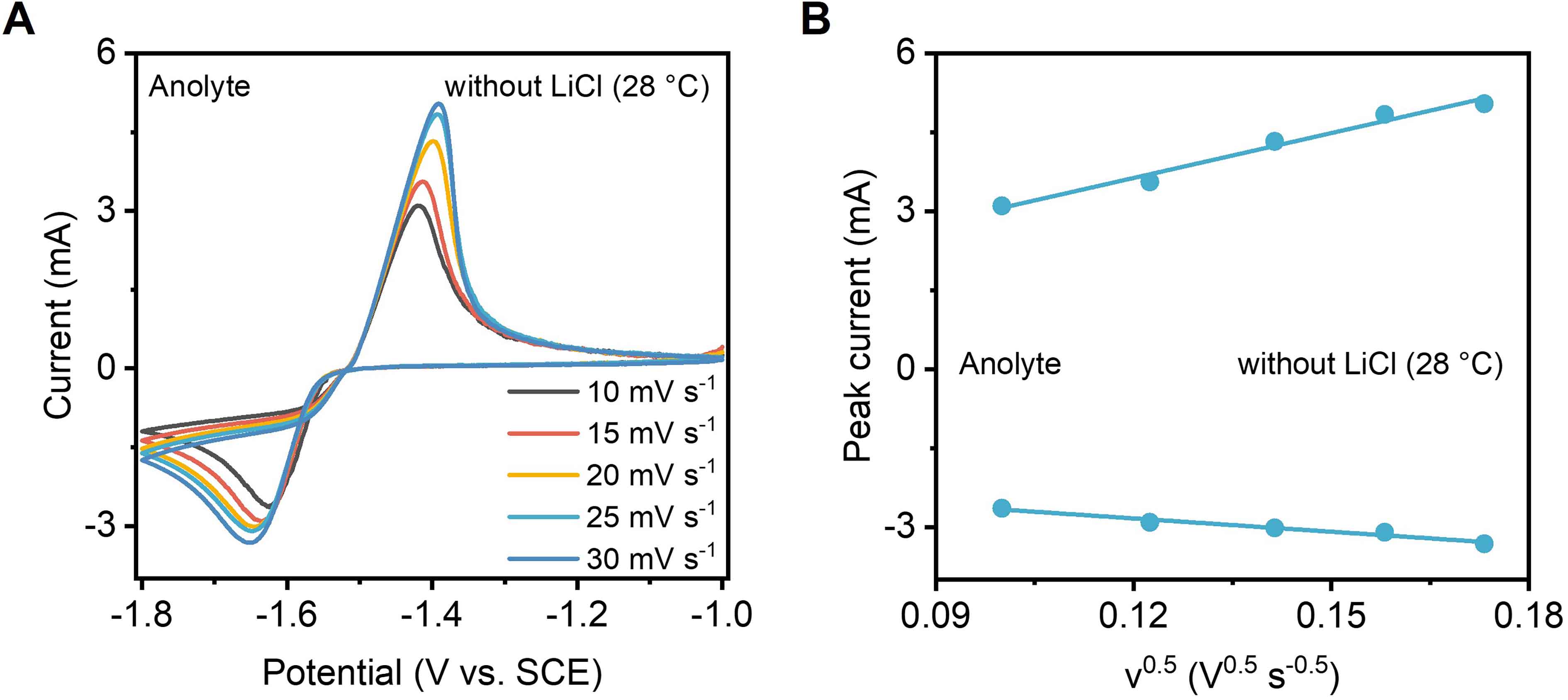


**Figure S24.** Electrochemical kinetics and diffusion-controlled behavior of the LiCl-free anolyte at 28 °C. (A) CV curves at different scan rates. (B) The linear relationship between the oxidation and reduction peak currents and the square root of the scan rate.


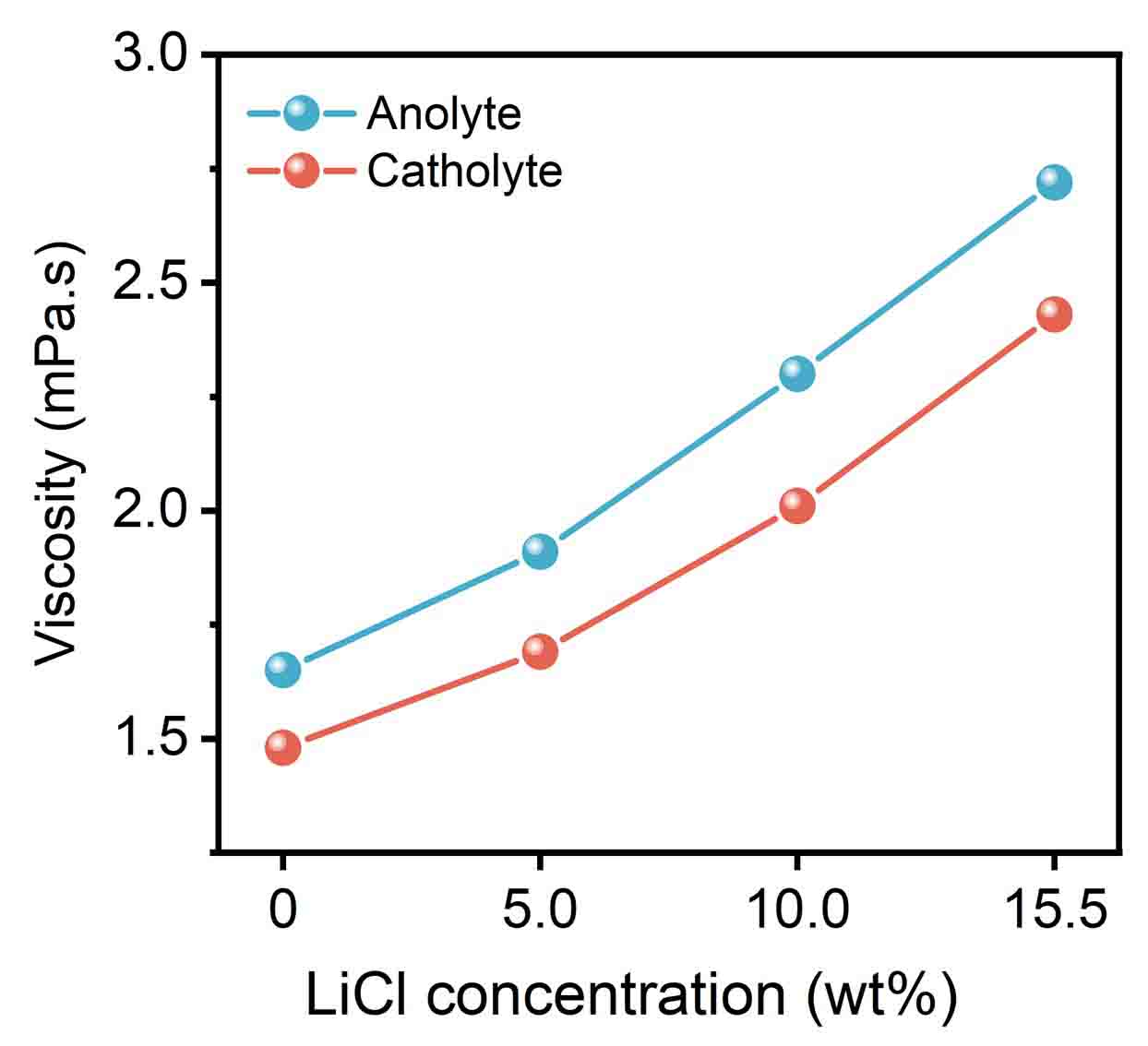


**Figure S25.** Viscosity values of the anolyte and catholyte vary with the concentration of LiCl.


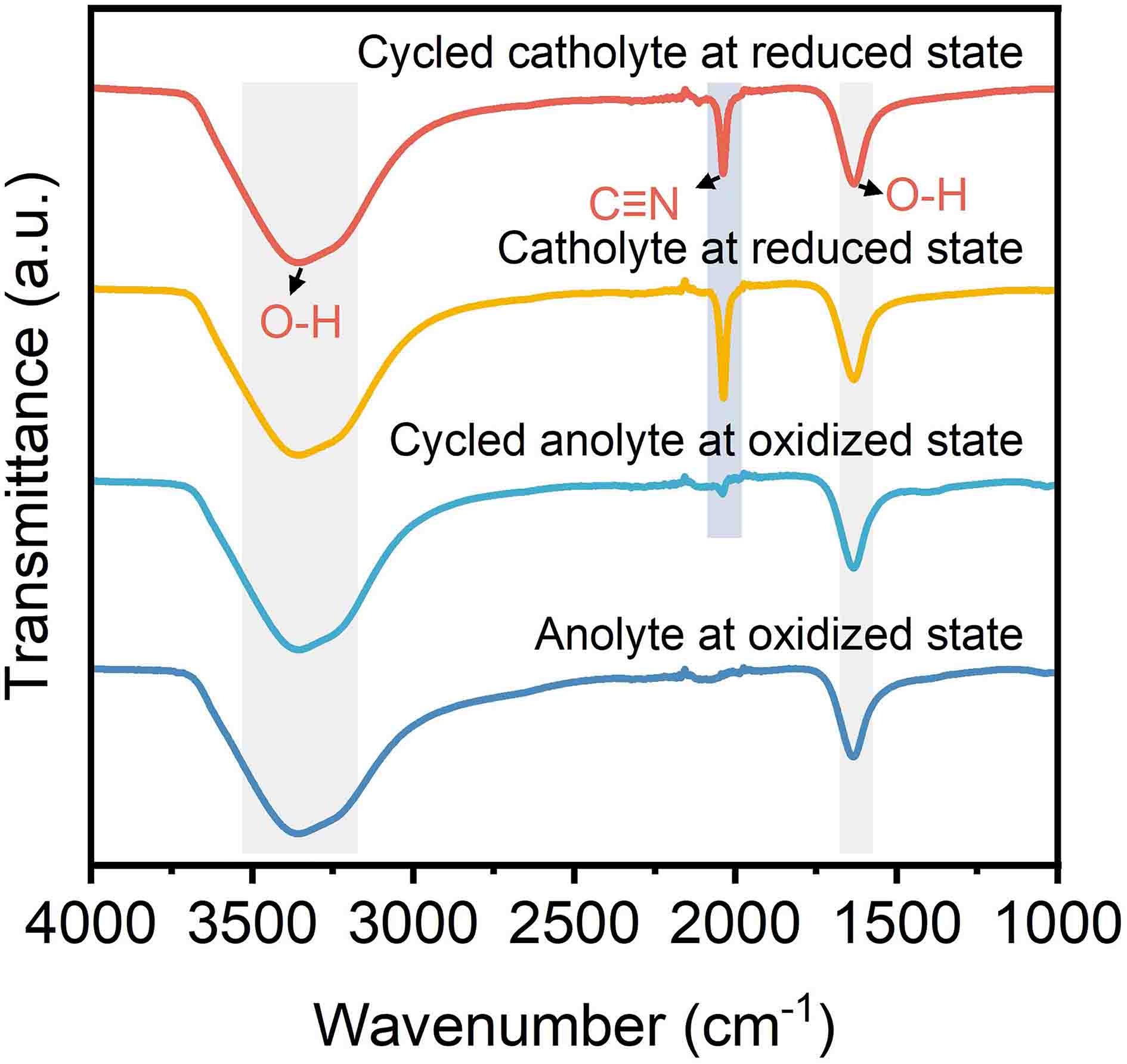


**Figure S26.** FTIR spectra of the reduced-state catholyte and the oxidized-state anolyte before and after 500 cycles.


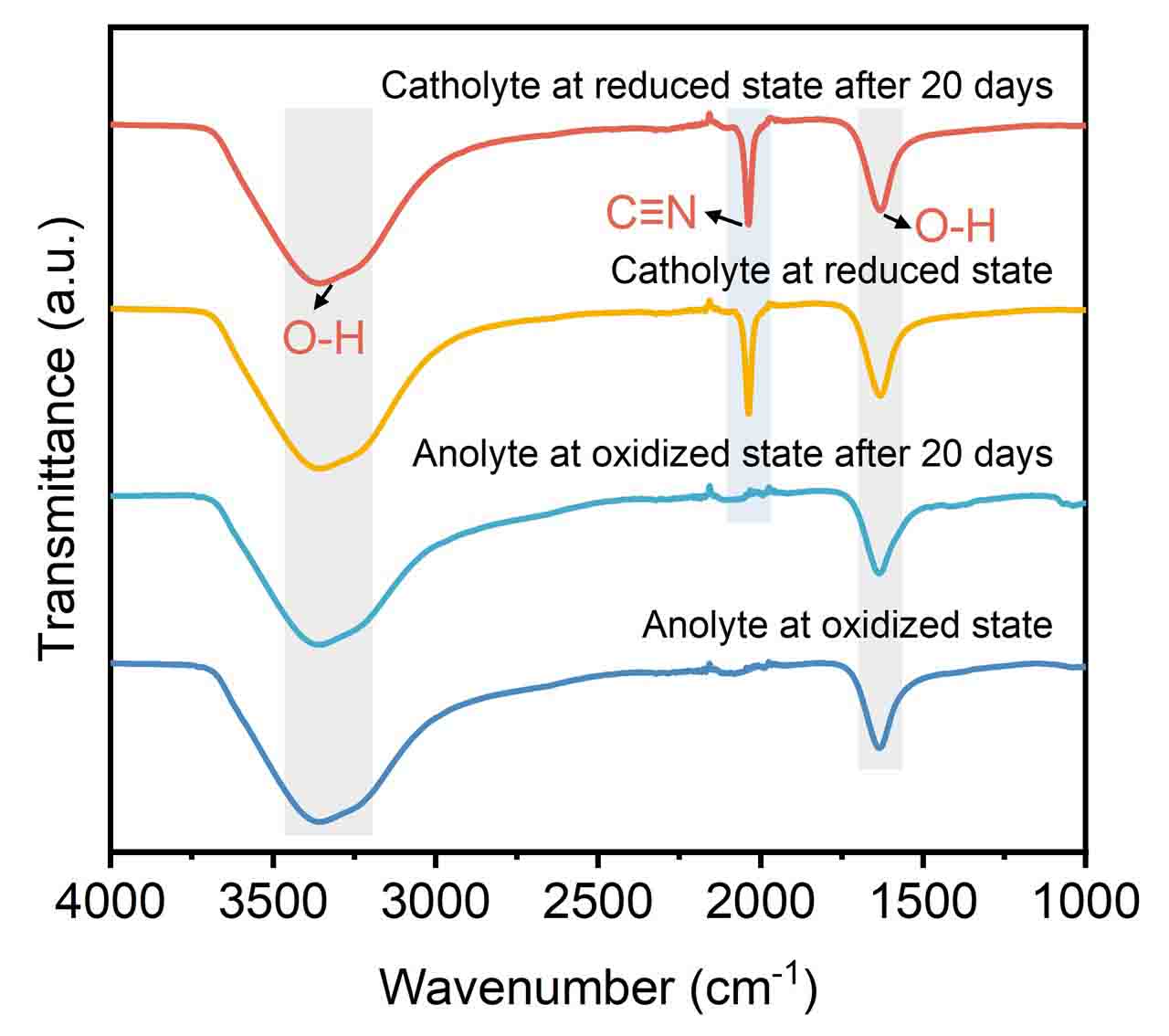


**Figure S27.** FTIR spectra of the reduced-state catholyte and the oxidized-state anolyte before and after 20-days of static storage.


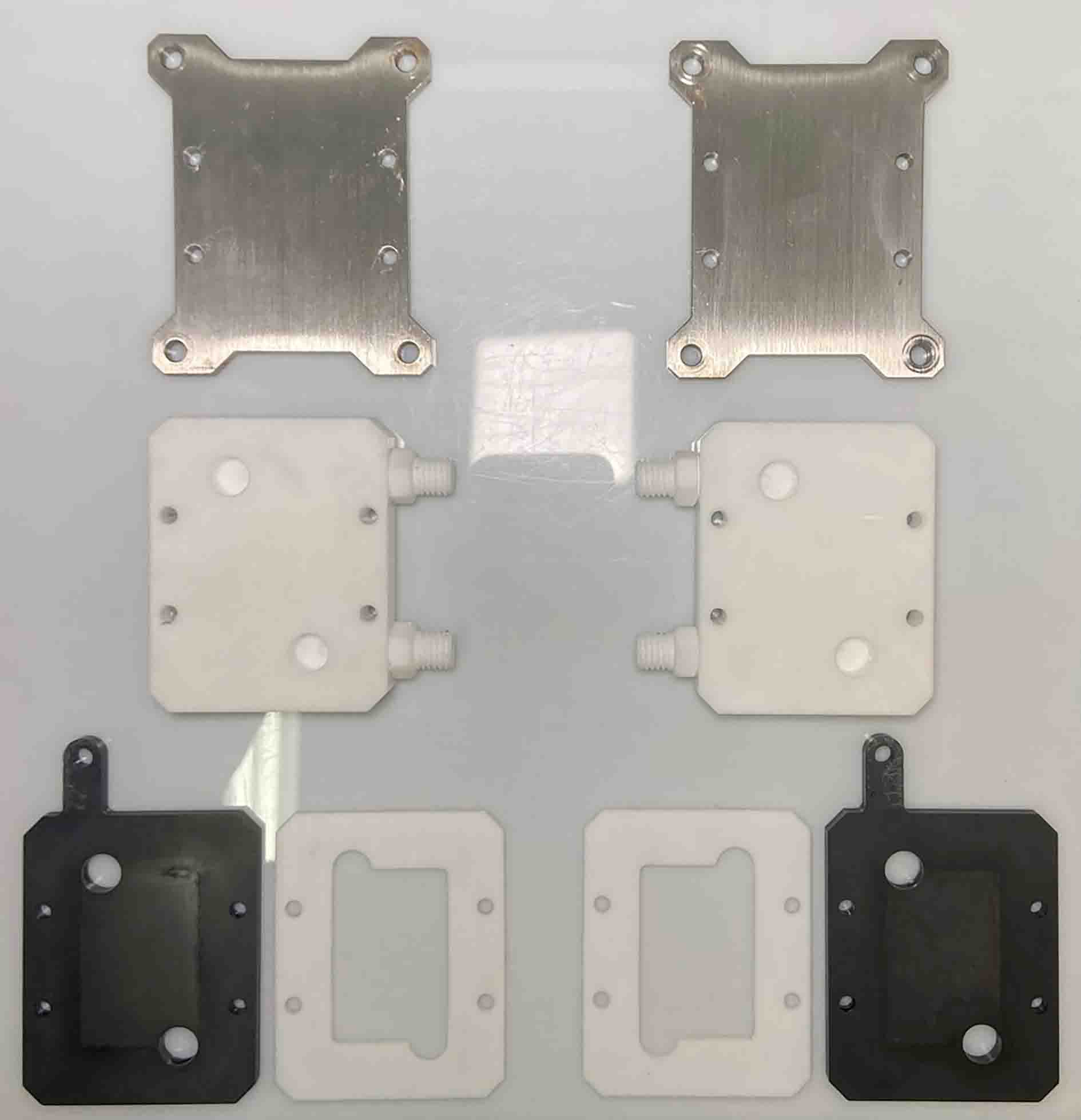


**Figure S28.** The structural diagram of the battery fixture without flow channels (active area: 4.5×3 cm^2^).


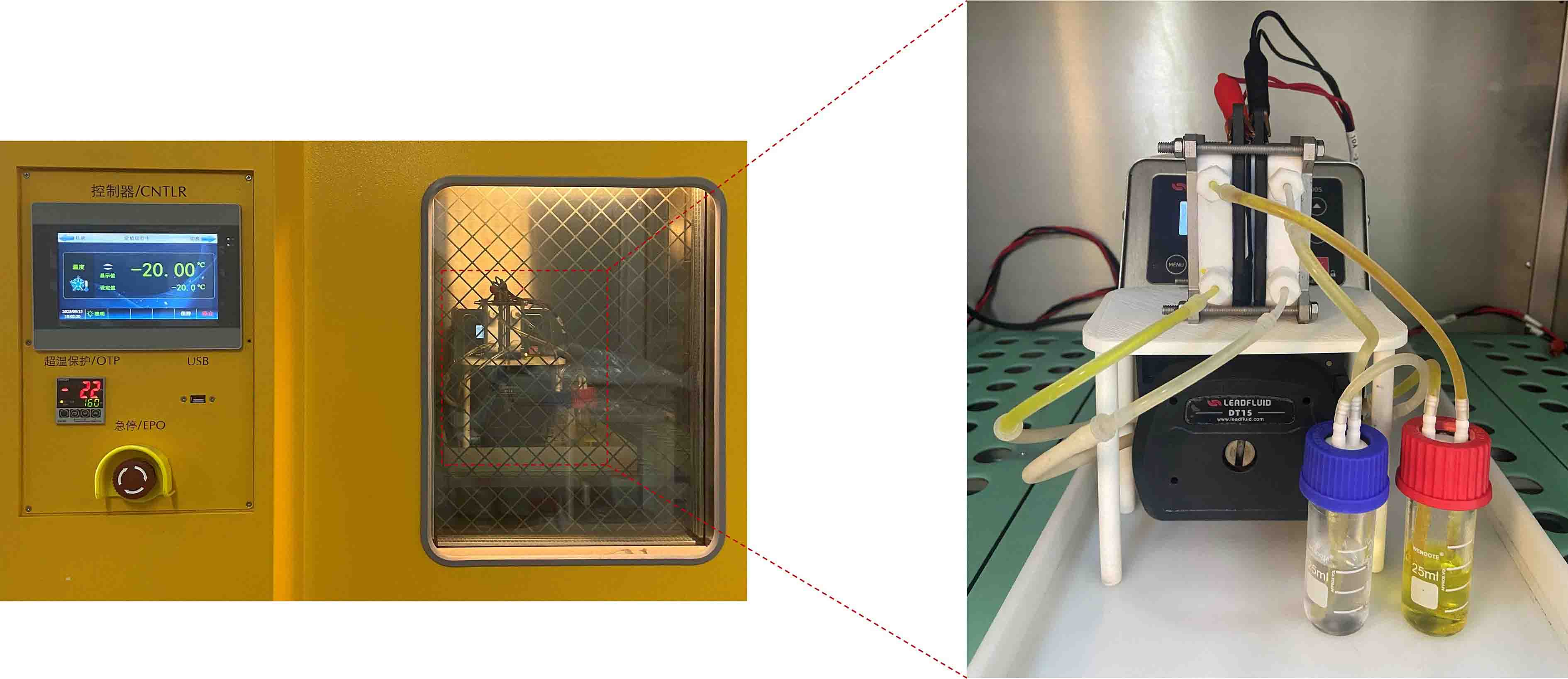


**Figure S29.** The physical picture of the flow battery operating at -20 °C (active area: 4.5×3 cm^2^).


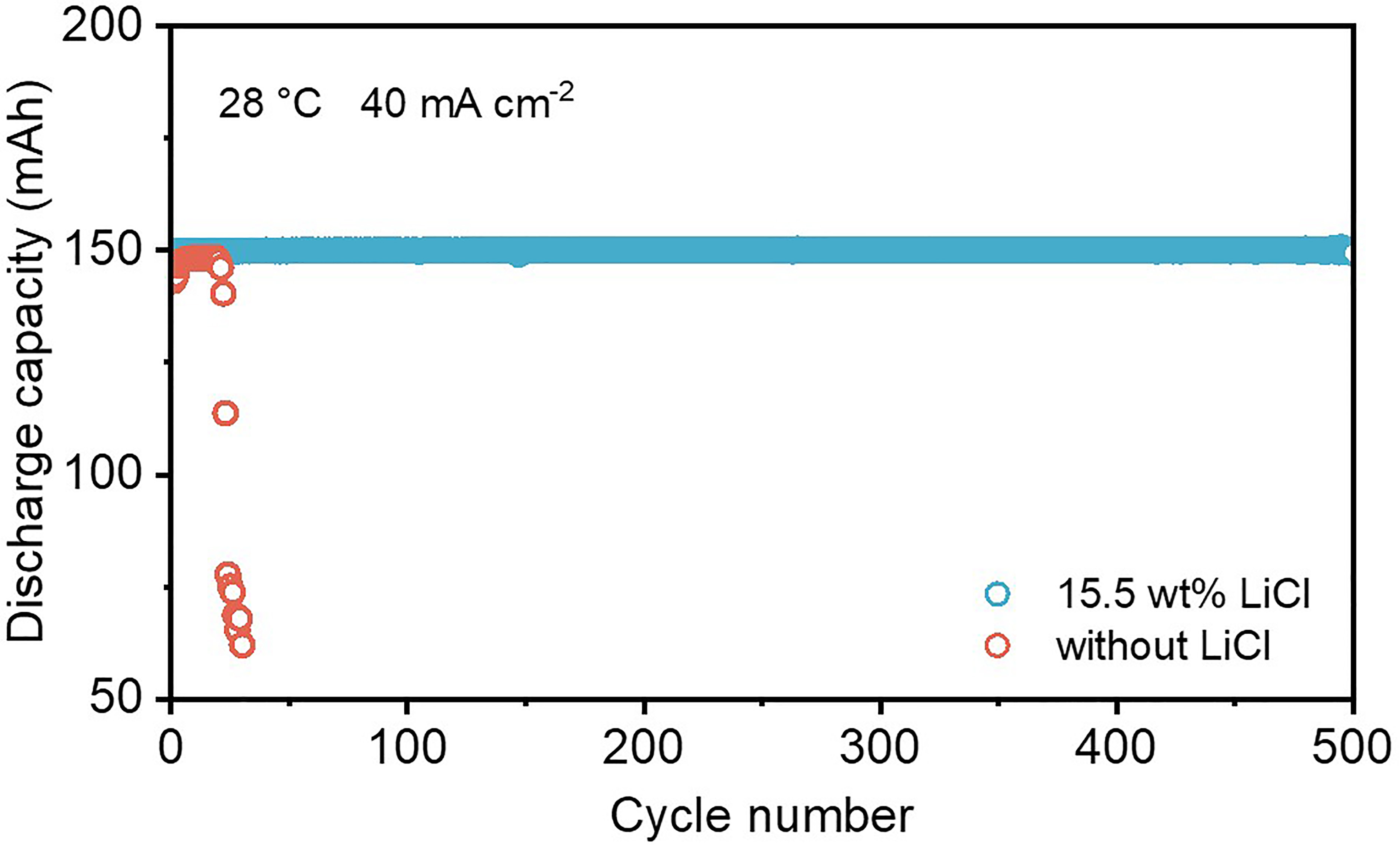


**Figure S30.** Discharge capacity of the AZFFB with and without LiCl at 28 °C and 40 mA cm^-2^ current density.


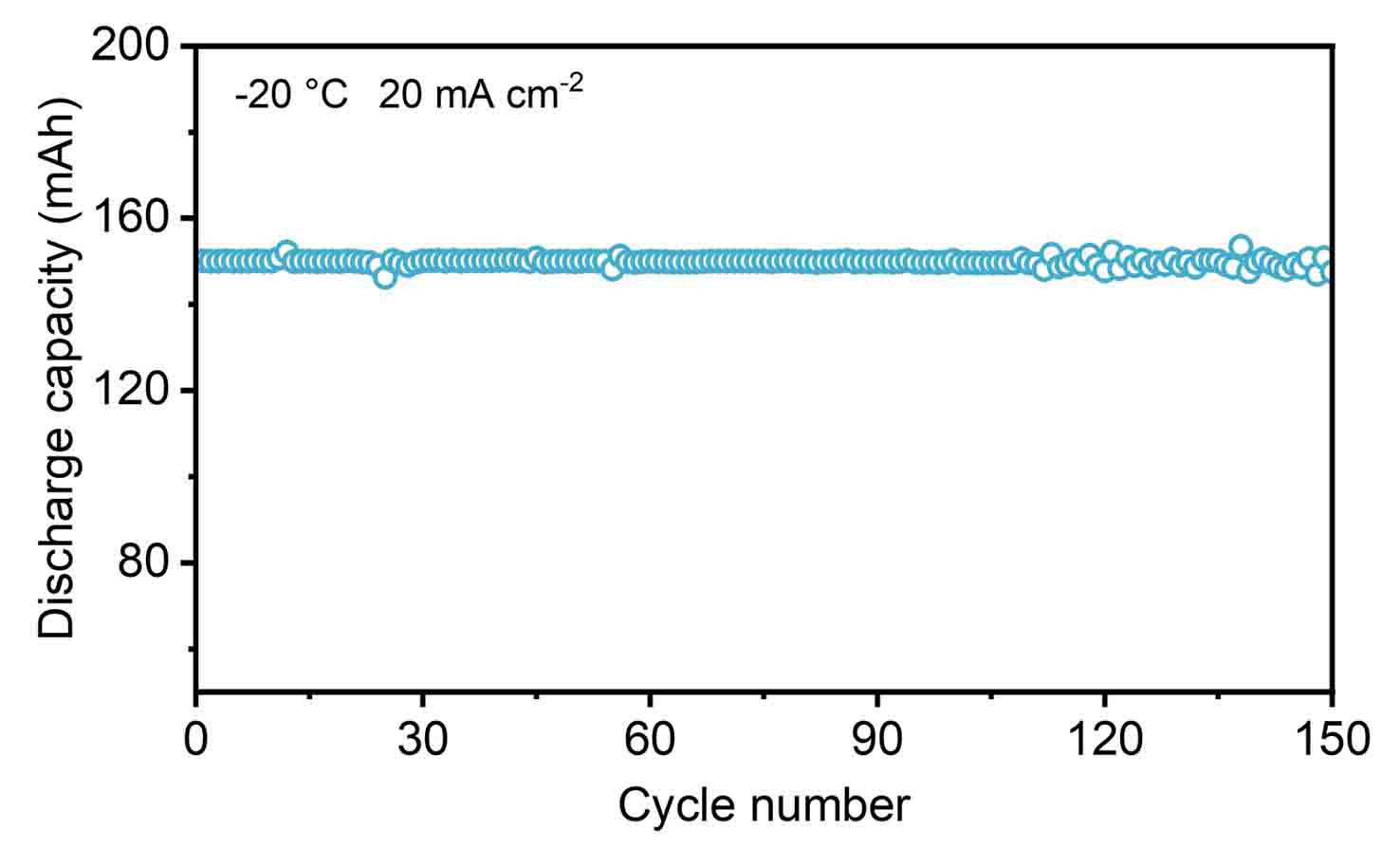


**Figure S31.** Discharge capacity of the LiCl-containing AZFFB at -20 °C and 20 mA cm^-2^ current density.


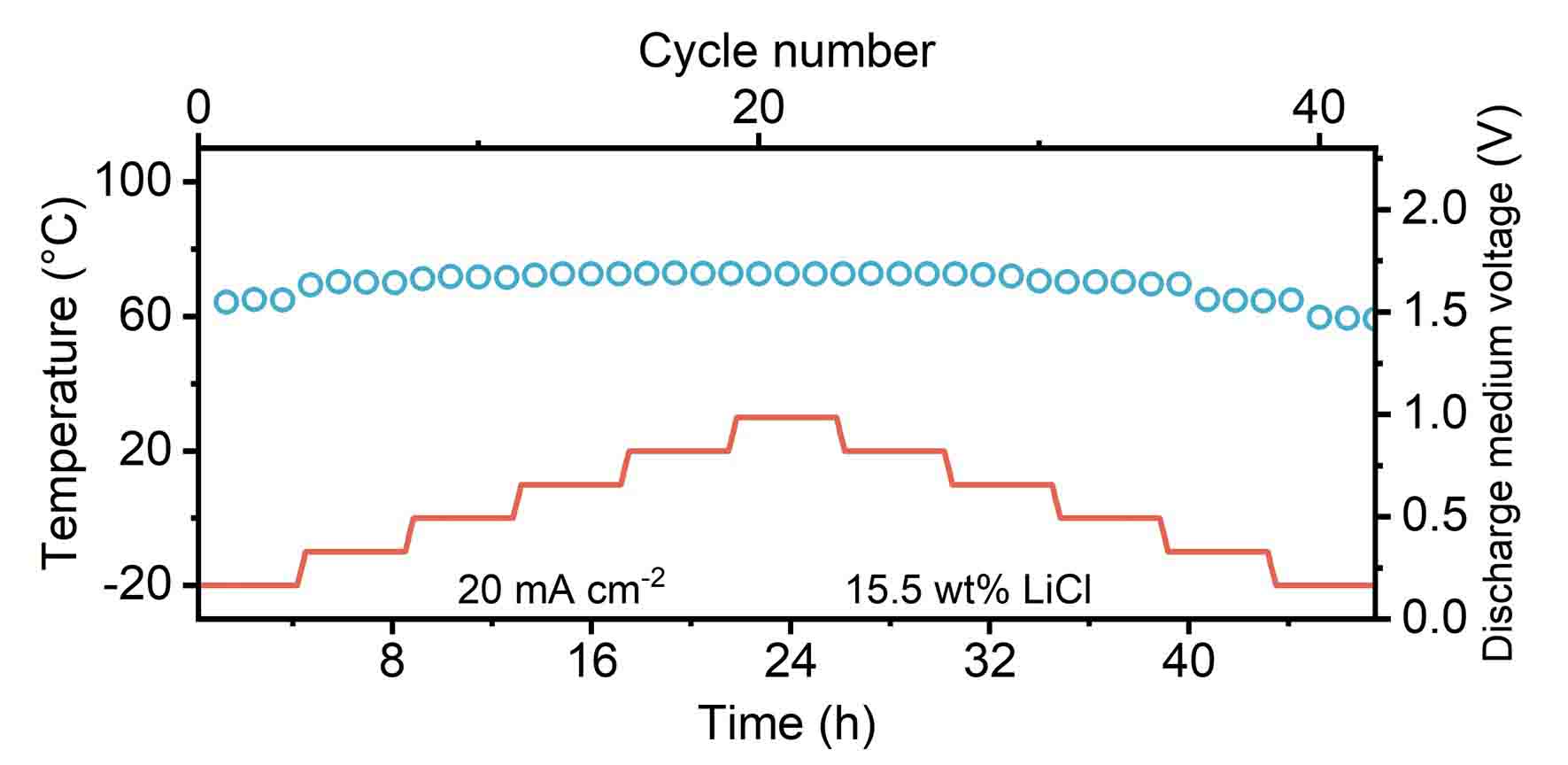


**Figure S32.** Discharge medium voltage of the AZFFB at variable temperature and 20 mA cm^-2^ current density.


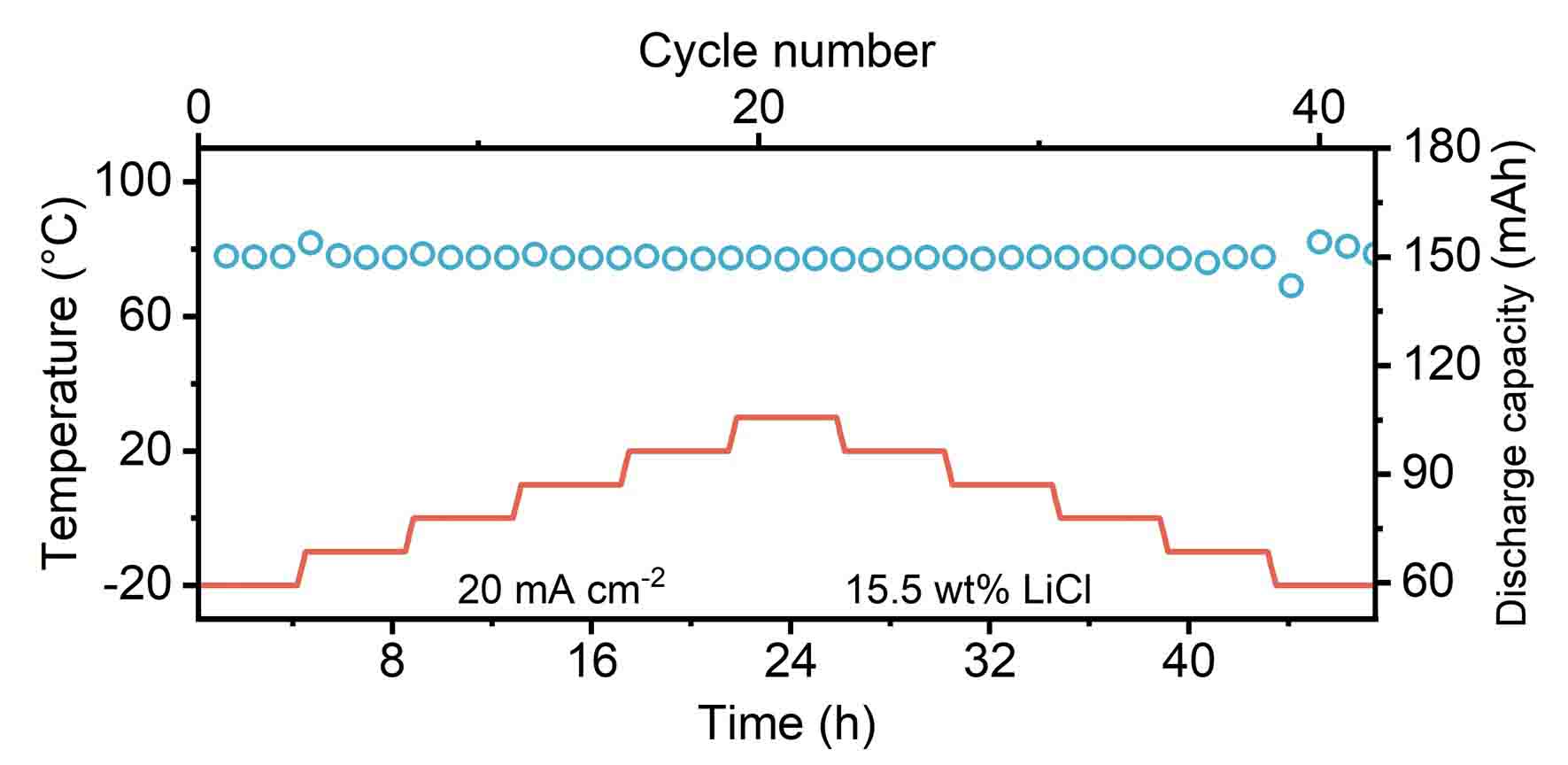


**Figure S33.** Discharge capacity of the AZFFB at variable temperature and 20 mA cm^-2^ current density.

**Supplementary Tables**

**Table S1.** A summary of MD simulations for three systems.

| System No. | Chemical substance | Unit cell edge length (Å) | Unit cell volume (Å³) | Chemical substance No. |
| --- | --- | --- | --- | --- |
| 1 | Zn(OH)_4_^2-^ | 40 | 64000 | 8 |
|  | Na^+^ | 40 | 64000 | 93 |
|  | OH^-^ | 40 | 64000 | 77 |
|  | Li^+^ | 40 | 64000 | 145 |
|  | Cl^-^ | 40 | 64000 | 145 |
|  | H_2_O | 40 | 64000 | 2000 |
| 2 | K^+^ | 40 | 64000 | 76 |
|  | Fe(CN)_6_^4-^ | 40 | 64000 | 19 |
|  | Na^+^ | 40 | 64000 | 19 |
|  | OH^-^ | 40 | 64000 | 19 |
|  | Li^+^ | 40 | 64000 | 145 |
|  | Cl^-^ | 40 | 64000 | 145 |
|  | H_2_O | 40 | 64000 | 2000 |
| 3 | K^+^ | 40 | 64000 | 76 |
|  | Fe(CN)_6_^4-^ | 40 | 64000 | 19 |
|  | Na^+^ | 40 | 64000 | 19 |
|  | OH^-^ | 40 | 64000 | 19 |
|  | H_2_O | 40 | 64000 | 2000 |

**Table S2.** MD simulation results of the number of water molecules around Fe(CN)_6_^4-^ with and without Li^+^ and Cl^-^.

|  | 0.5 M Fe(CN)_6_^4-^+15.5 wt% LiCl | | | 0.5 M Fe(CN)_6_^4-^ | | |
| --- | --- | --- | --- | --- | --- | --- |
| r (Å) | 2 | 4 | 6 | 2 | 4 | 6 |
| N (r) | 1.640 | 11.020 | 41.504 | 1.256 | 6.651 | 25.183 |

**Table S3.** Diffusion coefficients of the redox pairs of the catholyte and anolyte at different operating conditions.

| Electrolyte | Temperature (°C) | Diffusion coefficient (cm^2^ s^-1^) | |
| --- | --- | --- | --- |
|  |  | Oxidation state | Reduction state |
| Catholyte with LiCl | 28 | 9.39×10^-7^ | 9.17×10^-7^ |
| Anolyte with LiCl | 28 | 2.50×10^-6^ | 1.07×10^-7^ |
| Catholyte with LiCl | 0 | 6.58×10^-7^ | 6.62×10^-7^ |
| Anolyte with LiCl | 0 | 2.22×10^-7^ | 4.85×10^-8^ |
| Catholyte with LiCl | -20 | 2.44×10^-7^ | 2.03×10^-7^ |
| Anolyte with LiCl | -20 | 4.96×10^-8^ | 2.03×10^-8^ |
| Catholyte without LiCl | 28 | 1.56×10^-6^ | 1.52×10^-6^ |
| Anolyte without LiCl | 28 | 5.67×10^-6^ | 4.99×10^-7^ |

**Table S4.** Comparison of operating temperature and cycle numbers with existing research on zinc**–**ferricyanide flow batteries. Note: The room temperature in the reference was aggregated as 25 °C.

| Anolyte | Catholyte | Operating temperature (°C) | Coulombic efficiency (%) | Reference |
| --- | --- | --- | --- | --- |
| Zn/LiCl | Fe(CN)_6_^4-/3-^/LiCl | -20 | 99.54 | This work |
| Zn/LiCl | Fe(CN)_6_^4-/3-^/LiCl | 28 | 99.78 | This work |
| Zn/EDTA | Fe(CN)_6_^4-/3-^ | -10 | 99.2 | [16] |
| Zn/EDTA | Fe(CN)_6_^4-/3-^ | 60/0 | 98.1/99.7 | [17] |
| Zn | Fe(CN)_6_^4-/3-^ | 25 | 99.7 | [18] |
| Zn/urea | Fe(CN)_6_^4-/3-^ | 25 | 98.5 | [19] |
| Zn | Fe(CN)_6_^4-/3-^ | 25 | 99.2 | [20] |
| Zn | Fe(CN)_6_^4-/3-^ | 25 | 99.6 | [21] |
| Zn/DES | Fe(CN)_6_^4-/3-^ | 39.85 | 98.2 | [22] |
| Zn | Fe(CN)_6_^4-/3-^ | 50 | 98.5 | [23] |
| Zn/citrate | Fe(CN)_6_^4-/3-^ | 60 | 99 | [24] |

**References**

[1] Neese, F. An improvement of the resolution of the identity approximation for the formation of the Coulomb matrix. *J. Comput. Chem.* **2003**, *24*, 1740-1747.

[2] Neese, F. Software update: The ORCA program system—Version 5.0. *WIREs Comput. Mol. Sci.* **2022**, *12*, e1606.

[3] Neese, F.; Wennmohs, F.; Hansen, A.; Becker, U. Efficient, approximate and parallel Hartree–Fock and hybrid DFT calculations. A ‘chain-of-spheres’ algorithm for the Hartree–Fock exchange. *Chem. Phys.* **2009**, *356*, 98-109.

[4] Neese, F. The SHARK integral generation and digestion system. *J. Comput. Chem.* **2022**, *44*, 381-396.

[5] Grimme, S.; Antony, J.; Ehrlich, S.; Krieg, H. A consistent and accurate ab initio parametrization of density functional dispersion correction (DFT-D) for the 94 elements H-Pu. *J. Chem. Phys.* **2010**, *132*, 154104.

[6] Grimme, S.; Ehrlich, S.; Goerigk, L. Effect of the damping function in dispersion corrected density functional theory. *J. Comput. Chem.* **2011**, *32*, 1456-1465.

[7] Weigend, F.; Ahlrichs, R. Balanced basis sets of split valence, triple zeta valence and quadruple zeta valence quality for H to Rn: Design and assessment of accuracy. *Phys. Chem. Chem. Phys.* **2005**, *7*, 3297-3305.

[8] Lu, T. A comprehensive electron wavefunction analysis toolbox for chemists, Multiwfn. *J. Chem. Phys.* **2024**, *161*, 082503.

[9] Lu, T.; Chen, F. Multiwfn: A multifunctional wavefunction analyzer. *J. Comput. Chem.* **2011**, *33*, 580-592.

[10] Humphrey, W.; Dalke, A.; Schulten, K. VMD: Visual molecular dynamics. *J. Mol. Graph.* **1996**, *14*, 33-38.

[11] Spoel, D. V. D.; Lindahl, E.; Hess, B.; Groenhof, G.; Mark, A. E.; Berendsen, H. J. C. GROMACS: Fast, flexible, and free. *J. Comput. Chem.* **2005**, *26*, 1701-1718.

[12] Da Silva, A.W. S.; Vranken, W. F. ACPYPE - AnteChamber PYthon Parser interfacE. *BMC Res. Notes* **2012**, *5*, 367.

[13] Wei, B. Q.; Weaver, L. H.; Ferrari, A. M.; Matthews, B. W.; Shoichet, B. K. Testing a dlexible-receptor docking algorithm in a model binding site. *J. Mol. Biol.* **2004**, *337*, 1161-1182.

[14] Li, Z.; Song, L. F.; Li, P.; Jr, K. M. M. Systematic parametrization of divalent metal ions for the OPC3, OPC, TIP3P-FB, and TIP4P-FB water models. *J. Chem. Theory Comput.* **2020**, *16*, 4429-4442.

[15] Essmann, U.; Perera, L.; Berkowitz, M. L.; Darden, T.; Lee, H.; Pedersen, L.G. A smooth particle mesh Ewald method. *J. Chem. Phys.* **1995**, *103*, 8577-8593.

[16] Zhi, L.; Liao, C.; Xu, P.; Li, G.; Yuan, Z.; Li, X. Zinc-ferricyanide flow batteries operating stably under -10 °C. *Angew. Chem. Int. Ed.* **2024**, *63*, e202412559.

[17] Zhi, L.; Liao, C.; Xu, P.; Sun, F.; Fan, F.; Li, G.; Yuan, Z.; Li, X. New alkalescent electrolyte chemistry for zinc-ferricyanide flow battery. *Angew. Chem. Int. Ed.* **2024**, *63*, e202403607.

[18] Yu, D.; Zhi, L.; Zhang, F.; Song, Y.; Wang, Q.; Yuan, Z.; Li, X. Scalable alkaline zinc-iron/nickel hybrid flow battery with energy density up to 200 Wh L^-1^. *Adv. Mater.* **2023**, *35*, 2209390.

[19] Ling, R.; Zhu, Z.; Peng, K.; Fang, J.; Zou, W.; Li, Q.; Liu, Y.; Zhu, Q.; Lin, N.; Xu, T.; Yang, Z. Dual-function electrolyte additive design for long life alkaline zinc flow batteries. *Adv. Mater.* **2024**, *36*, 2404834.

[20] Yang, X.; Wei, Z.; Hu, H.; Hong, H.; Wang, Y.; Zhu, J.; Li, P.; Wang, T.; Fan, J.; Zhi, C. Lewis acid-driven weak electrostatic interaction of polybenzimidazole-based membrane for alkaline zinc-iron redox flow batteries, *Adv. Mater*. **2025**, e10023. DOI: 10.1002/adma.202510023

[21] Wang, S.; Li, T.; Yuan, C.; Zhu, J.; Li, P.; Zhang, S.; Wei, Z.; Wang, Y.; Li, X.; Zhi, C. Realizing an anolyte utilization rate of 99% in low-cost zinc-based flow batteries by rejuvenating dead zinc, *Energy Environ. Sci*. **2024**, *17*, 7155.

[22] Cao, X.; Wang, X.; Xue, X. A Low-Cost and Green Zinc-Iron Battery Achieved by Ethaline Deep Eutectic Solvent. *ChemSusChem* **2024**, *18*, e202401604.

[23] Hu, J.; Yue, M.; Zhang, H.; Yuan, Z.; Li, X. A Boron Nitride Nanosheets Composite Membrane for a Long-Life Zinc-Based Flow Battery. *Angew. Chem., Int. Ed.* **2020**, *59*, 6715-6719.

[24] Chen, Z.; Li, T.; Xie, C.; Li, X. A neutral zinc–iron flow battery with long lifespan and high power density. *ACS Energy Lett.* **2024**, *9*, 3426-3432.
